# Supplementary material for: Loss of the mitochondrial i‐AAA protease YME1L leads to ocular dysfunction and spinal axonopathy
Source: EMBO Mol Med. 2018 Nov 2;11(1):e9288. doi: 10.15252/emmm.201809288 (PMC6328943; doi:10.15252/emmm.201809288)
Supplement: Supplementary file 1 — Appendix [file EMMM-11-e9288-s001.pdf]

APPENDIX Sprenger et al

Table of Contents

1) **Appendix Figure Legends**
2) **Appendix Figure S1**
3) **Appendix Figure S2**
4) **Appendix Figure S3**
5) **Appendix Figure S4**
6) **Appendix Figure S5**
7) **Appendix Figure S6**
8) **Appendix Methods**
9) **Appendix Primer Sequences**
10) **Appendix Statistical Analyses**

Appendix Figure Legends

Appendix Figure S1 - Microphthalmia in NYKO mice is independent of ROS and Caspase 9 activation.

A Quantification of nuclei (area = 1000 μm^2) in inner nuclear (INL) and outer nuclear layer (ONL) of 6-7 week old WT (n = 3) and NYKO (n = 3) mice.

B mtDNA levels (*Cytb*/*Actin*) in retinas of 6-7 week old WT (n = 5) and NYKO (n = 5) mice.

C mRNA levels in retinas of 6-7 week old WT (n = 5) and NYKO (n = 5) mice. Transcript levels were normalized to *Hprt* mRNA levels.

D Immunoblot analysis of retinas isolated from 6-7 week old WT and NYKO mice. Antibodies against SDHA were used to control for gel loading.

E mRNA levels in retinas of 6-7 week old WT (n = 5) and NYKO (n = 5) mice. Transcript levels were normalized to *Hprt* mRNA levels.

F Immunoblot analysis of retinas isolated from 6-7 week old WT and NYKO mice. Antibodies against SDHA were used to control for gel loading.

G Optic nerve mean g-ratios of 367 axons from three different WT animals and 353 axons from three different NYKO animals

Unpaired t-test, $^*P \leq 0.05$, ns = not significant. Data are means ± SEM.

Appendix Figure S2 - Absence of neuroinflammation in the brain of NYKO mice.

A Representative immunohistochemical stainings of sagittal sections across different brain regions from 31-32 week old WT and NYKO mice using IBA1-, and Calbindin-specific antibodies. Scale bars 100 µm.

B mRNA levels of proinflammatory cytokines in cerebella of 31-32 week old WT (n = 4) and NYKO mice (n = 5). Transcript levels were normalized to *Hprt* mRNA levels.

Unpaired t-test, ns = not significant. Data are means ± SEM.

Appendix Figure S3 - Validation of *Yme1l* knockout in cultured cortical neurons.

PCRs amplifying wildtype (WT = 241 bp), loxP flanked (loxP = 287 bp) or recombined (deleted = 303 bp) *Yme1l* alleles. Genomic DNA isolated from cultured cortical neurons ($Yme1l^{fl/fl}$) after transfection with CAG-GFP or CAG-Cre-IRES-GFP expressing plasmids, respectively.

Appendix Figure S4 - Impaired OXPHOS supercomplex formation in aged NYKO mice.

A Immunoblot analysis of spinal cord mitochondria isolated from 6-7 week old WT and NYKO mice using antibodies directed against OXPHOS subunits.

B Blue native (BN-) PAGE followed by immunoblotting of spinal cord mitochondria isolated from 6-7 week old WT (n = 3) and NYKO (n = 3) mice using the indicated antibodies. Mitochondrial membranes were solubilized with n-dodecyl-D-maltoside (DDM, 2.5 g/g protein). CI signal on UQCRC2 and ATP5α blots present from previous decoration with NDUFA9.

C, D BN-PAGE followed by immunoblot analysis of isolated spinal cord mitochondria from 31-32 week old WT (n = 5) and NYKO (n = 5) mice using indicated antibodies. Mitochondrial membranes were solubilized with digitonin (6 g/g protein).

Unpaired t-test, *$P \leq 0.05$, **$P \leq 0.01$. Data are means ± SEM.

Appendix Figure S5 - Brain mitochondria are fragmented in NYKO mice.

A Mitochondrial network analysis in cerebella of 31-32 week old WT (n = 6) and NYKO mice (n = 7) using TOMM20-specific antibodies. Scale bars 10 µm. Unpaired t-test, ****$P \leq 0.0001$. Data are means ± SEM.

B, **C** Immunoblot analysis of spinal cord lysates from 6-7 week old WT and NYKO mice using the indicated antibodies. In b short and long exposure of the same OPA1 antibody decoration are shown. SDHA, HSP60 and ACTIN were used to control for gel loading.

Appendix Figure S6 – Mitochondrial ultrastructure, brain morphology and autophagy in NYOKO mice.

A, B TEM of dorso-lateral tracts of spinal cords from 6-7 week old NYOKO mice (n = 3, 744 mitochondria). Scale bars 200 nm.

C Nissl stainings of sagittal sections across different brain regions of 6-7 week old WT (n = 3), NOKO (n = 3), and NYOKO (n = 3) mice. Scale bars 4x = 500 µm; 40x = 50 µm.

D Immunoblots of 6-7 week old spinal cord lysates. TUBULIN was used to control for gel loading.

E mRNA levels of autophagy related genes from 6-7 week old spinal cords (WT, n = 5; NYOKO, n = 5). Transcript levels were normalized to *Hprt* mRNA levels.

F Immunoblots of 6-7 week old spinal cord lysates. Marker proteins for different mitochondrial compartments were used: TOMM20 = outer mitochondrial membrane, ATP5A = inner mitochondrial membrane, LONP1 = mitochondrial matrix. ACTIN was used to control for gel loading.

Unpaired t-test, ns = not significant. Data are means ± SEM.

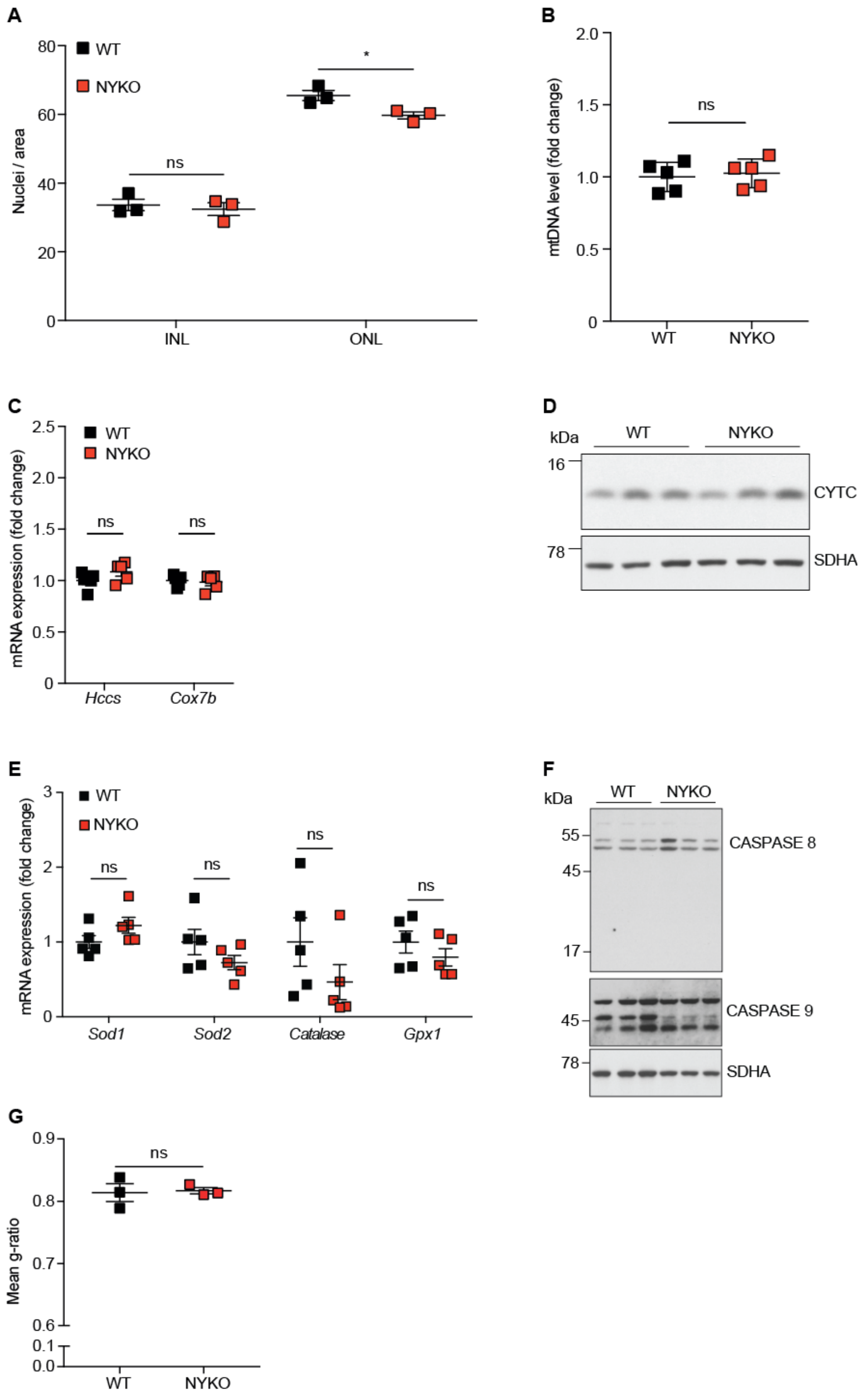

Appendix Fig S1

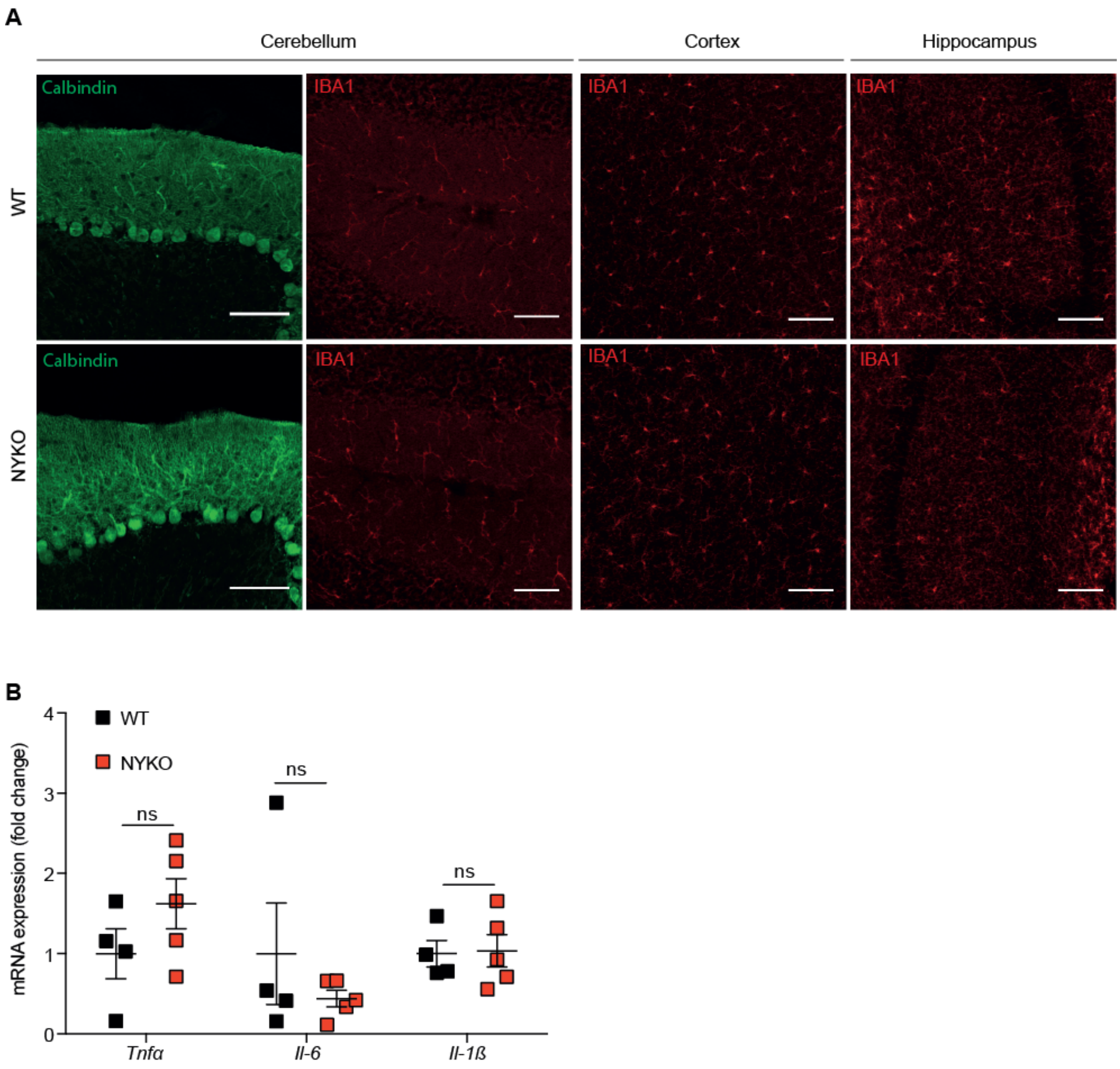

Appendix Fig S2

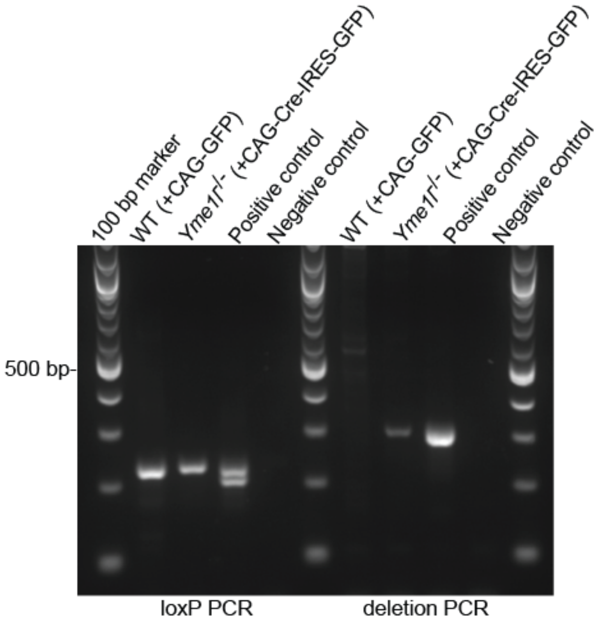

Appendix Fig S3

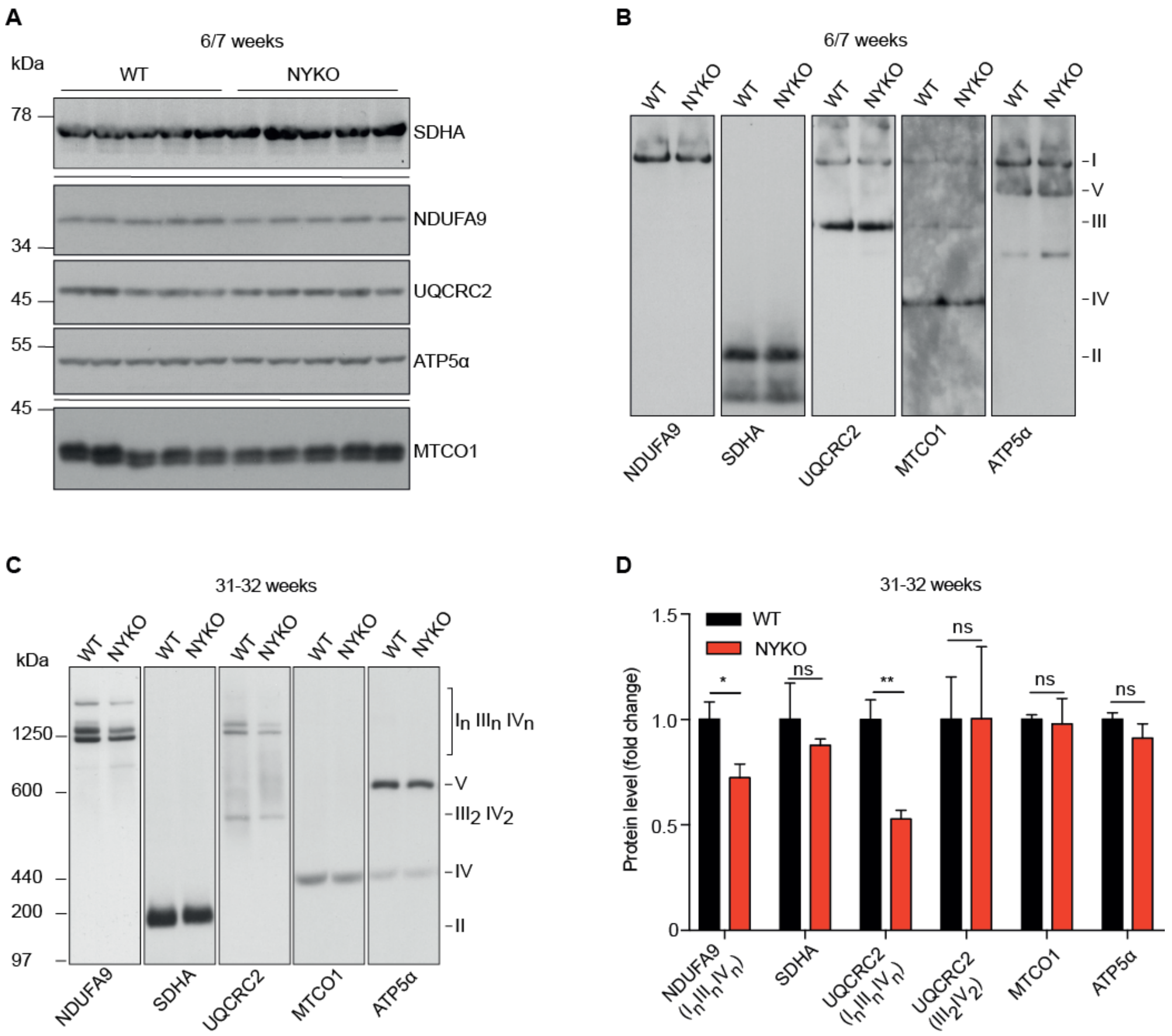

Appendix Fig S4

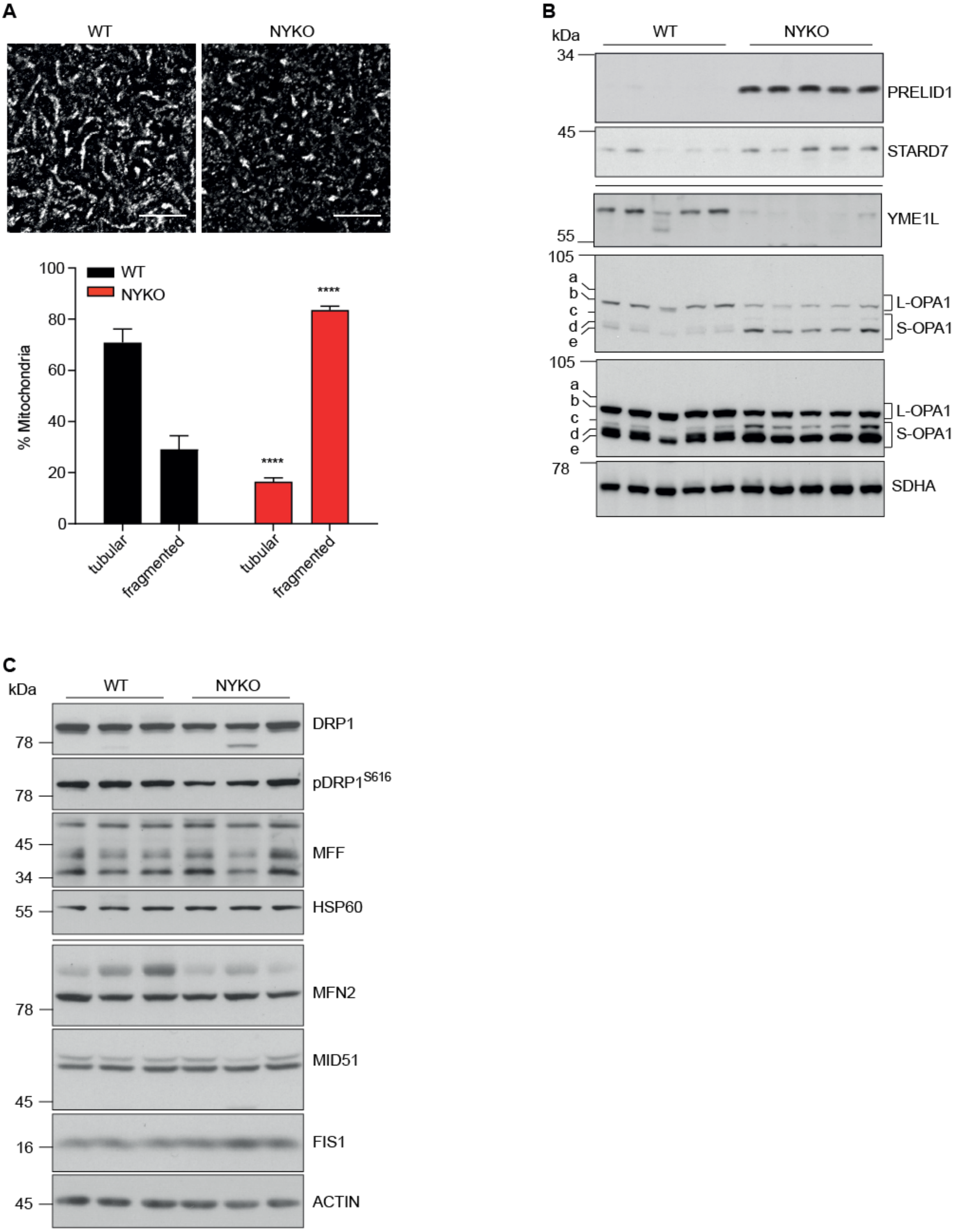

Appendix Fig S5

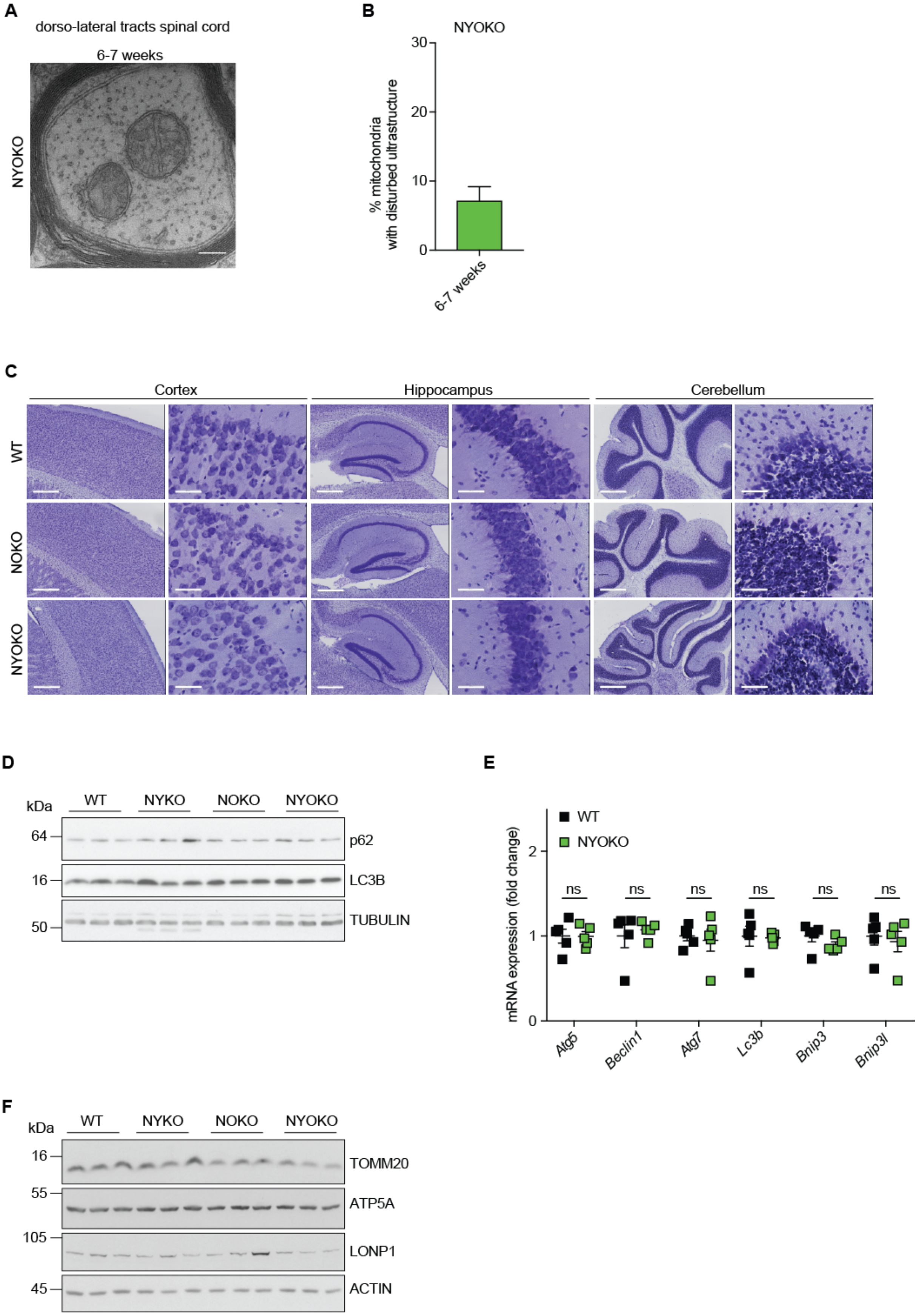

Appendix Fig S6

Appendix Methods

mtDNA isolation

Total DNA was isolated from snap-frozen retinas by standard proteinase K and phenol-chloroform methods. MtDNA copy number levels were analyzed by quantitative real-time PCR. The amplification levels of the mitochondrial CytB gene were normalized against the nuclear beta-actin gene.

Appendix Primer Sequences

Complementary DNA primer:

Tnfα fwd: CTTCTGTCTACTGAACTTCGGG

Tnfα rev: CAGGCTTGTCACTCGAATTTTG

Il-6 fwd: TAGTCCTTCCTACCCCAATTTCC

Il-6 rev: TTGGTCCTTAGCCACTCCTTC

Il-1β fwd: GAAATGCCACCTTTTGACAGTG

Il-1β rev: TGGATGCTCTCATCAGGACAG

S100a9 fwd: TGAGCAAGAAGGAATTCAGACAAA

S100a9 rev: TGTGTCCAGGTCCTCCATGA

Asc fwd: GAAGCTGCTGACAGTGCAAC

Asc rev: GCCACAGCTCCAGACTCTTC

Rage fwd: GAAGGCTCTGTGGGTGAGTC

Rage rev: CCGCTTCCTCTGACTGATTC

Hmgb1 fwd: CGCGGAGGAAAATCAACTAA

Hmgb1 rev: TCATAACGAGCCTTGTCAGC

Myd88 fwd: GAAACTCCACAGGCGAGCGTA

Myd88 rev: GTTAAGCGCGACCAAGGGTATG

Fgf21 fwd: ATGGAATGGATGAGATCTAGAGTTGG

Fgf21 rev: TCTTGGTGGTCATCTGTGTAGAGG

Hccs fwd: GCCATTTACTTTGTCTACCTC

Hccs rev: CATCATCTTTCCACTTCCAC

Cox7b fwd: TCTACATGGACATATACAGCC

Cox7b rev: ACATGATTCTTTGACTTGGC

Sod1 fwd: CAAGCGGTGAACCAGTTGTG

Sod1 rev: TGAGGTCCTGCACTGGTAC

Sod2 fwd: GCCTGCACTGAAGTTCAATG

Sod2 rev: ATCTGTAAGCGACCTTGCTC

Catalase fwd: ACCCTCTTATACCAGTTGGC

Catalse rev: GCATGCACATGGGGCCATCA

Gpx1 fwd: GTCTCTCTGAGGCACGATCCG

Gpx1 rev: TTCCGCAGGAAGGTAAACAGC

Atg5 fwd: GACCACAAGCAGCTCTGGAT

Atg5 rev: GGTTTCCAGCATTGGCTCTA

Beclin1 fwd: TGATCCAGGAGCTGGAAGAT

Beclin1 rev: CAAGCGACCCAGTCTGAAAT

Atg7 fwd: TCCGTTGAAGTCCTCTGCTT

Atg7 rev: CCACTGAGGTTCACCATCCT

Lc3b fwd: CCGAGAAGACCTTCAAGCAG

Lc3b rev: ACACTTCGGAGATGGGAGTG

Bnip3 fwd: AGCTTTGGCGAGAAAAACAG

Bnip3 rev: TCCAATGTAGATCCCCAAGC

Bnip3l fwd: AACAACAACTGCGAGGAAGG

Bnip3l rev: GTCCCTGCTGGTATGCATCT

Genomic DNA primer:

Cytb fwd: GCTTTCCACTTCATCTTACCATTT

Cytb rev: TGTTGGGTTGTTTGATCCTG

Actin fwd: GGAAAAGAGCCTCAGGGCAT

Actin rev: GAAGAGCTATGAGCTGCCTGA

Yme1l loxP fwd: TTGAACTCAGAGATTGGCCTGTC

Yme1l loxP rev: CTGGGACAAACACTTTGGATG

Yme1l deletion fwd: GTTTCTCTGTGTATCCCTGGTTGTC

Yme1l deletion rev: CCAAGTGAAGCAAGTGCCTCACGAT

Appendix Statistical Analyses

Fig.1C t: 12.41, degrees of freedom: 3.455, *p* value: 0.0005 **Fig. 1E** t ratio: *Tnf*α = 8.02, *Il-6* = 1.806, *Il-1*β = 6.453, *S100a9* = 1.771, *Asc* = 8.389, *Rage* = 0.04448, *Hmgb1* = 2.153, *Myd88* = 2.569, *p* value: *Tnf*α = 0.000043, *Il-6* = 0.109 *Il-1*β = 0.0002, *S100a9* = 0.1145, *Asc* = 0.000031, *Rage* = 0.9656, *Hmgb1* = 0.06347, *Myd88* = 0.0332, degrees of freedom: 8, **Fig. 1F** t ratio: 3.717, *p* value: 0.0059, degrees of freedom: 8 **Fig. 2B** t ratio: 11.2, *p* value: 0.0000000029, degrees of freedom: 17, **Fig. 2D** t: 6-7 weeks = 0.2256, 17-18 weeks = 5.578, *p* value: 6-7 weeks = 0.8241, 17-18 weeks = <0.0001, degrees of freedom: 6-7 weeks = 18, 17-18 weeks = 15, **Fig. 2D** f value: 18.72, *p* value: <0.0001, degrees of freedom: 39, **Fig. 2E** t: 6-7 weeks = 0.08818, 17-18 weeks = 2.535, *p* value: 6-7 weeks = 0.9307, 17-18 weeks = 0.0229, degrees of freedom: 6-7 weeks = 18, 17-18 weeks = 15, **Fig. 2E** f value: 55.06, *p* value: <0.0001, degrees of freedom: 39, **Fig. 3A** t: 1.501, *p* value: 0.1458, degrees of freedom: 25.12, **Fig. 3C** t ratio: Cortex = 0.8935, Hippocampus = 1.136, Cerebellum = 0.9246, *p* value: Cortex = 0.3926, Hippocampus = 0.2826, Cerebellum = 0.3769, degrees of freedom: 10 **Fig. 4C** t ratio: 6-7 weeks (cervical) = 0.5648, 6-7 weeks (lumbar) = 0.9871, 17-18 weeks (cervical) = 7.712, 17-18 weeks (lumbar) = 3.16, 31-32 weeks (cervical) = 6.163, 31-32 weeks (lumbar) = 5.478, *p* value: 6-7 weeks (cervical) = 0.5927, 6-7 weeks (lumbar) = 0.3617, 17-18 weeks (cervical) = 0.00025, 17-18 weeks (lumbar) = 0.0196, 31-32 weeks (cervical) = 0.000166, 31-32 weeks (lumbar) = 0.000192, degrees of freedom: 6-7 weeks = 6, 17-18 weeks = 6, 31-32 weeks (cervical) = 9, 31-32 weeks (lumbar) = 11, **Fig. 4E** t ratio: *Tnf*α = 8.303, *Il-6* = 1.559, *Il-1*β = 5.322, *p* value: *Tnf*α = 0.0000334, *Il-6* = 0.1577, *Il-1*β = 0.00071, degrees of freedom: 8, **Fig. 5B** t: 0.8659, *p* value: 0.3951, degrees of freedom: 24, **Fig. 5C** t: 2.67, *p* value: 0.0137, degrees of freedom: 23, **Fig. 5D** t: 0.9164, *p* value: 0.3690, degrees of freedom: 23, **Fig. 5E** t: 1.623, *p* value: 0.1182, degrees of freedom: 23 **Fig. 5G** t ratio: anterograde = 4.261, retrograde = 0.5295, *p* value: anterograde = 0.0469, retrograde = 0.6245, degrees of freedom = 4, **Fig. 5I** t ratio: moving mitochondria = 3.206, *p* value: moving mitochondria = 0.0327, degrees of freedom: moving mitochondria = 4, **Fig. 5J** t: 4.299, *p* value: 0.0127, degrees of freedom: 4, **Fig. 6A** t ratio: CI = 0.7376, CI + CII = 1.002, State 4 = 0.1536, Uncoupled = 0.8125, *p* value: CI = 0.4679, CI + CII = 0.3261, State 4 = 0.8792, Uncoupled = 0.4245, degrees of freedom: 24, **Fig. 6B** t ratio: CI = 3.17, CI + CII = 3.877, State 4 = 0.3449, Uncoupled = 3.6, *p* value: CI = 0.00412, CI + CII =

0.00072, State 4 = 0.7332, Uncoupled = 0.00144, degrees of freedom: 24, **Fig. 6D** t ratio: 6-7 weeks = 0.3835, 17-18 weeks = 6.117, 31-32 weeks = 3.536, *p* value: 6-7 weeks = 0.7146, 17-18 weeks = 0.000872, 31-32 weeks = 0.0241, degrees of freedom: 6-7 weeks = 6, 17-18 weeks = 6, 31-32 weeks = 4, **Fig. 7B** Kruskal-Wallis test, number of groups = 3 (WT, NYKO and NYOKO), Kruskal-Wallis statistic = 12.9, *p* value: WT vs. NYKO = 0.0488, WT vs. NYOKO = 0.8130 **Fig. 7D** Mann-Whitney test, Mann-Whitney U = 16685, *p* value: <0.0001, **Fig. 8B** see Fig. 7b, **Fig. 8F** f value: cervical = 489.8, lumbar = 51.14, *p* value: cervical = WT vs. NOKO = 0.1259, WT vs NYOKO = < 0.0001, NOKO vs. NYOKO = < 0.0001, lumbar = WT vs. NOKO = 0.9938, WT vs. NYOKO = 0.0003, NOKO vs. NYOKO = 0.0003, degrees of freedom: 6, **Fig. 8G** f value: 36.91, *p* value: WT vs. NOKO = >0.9999, WT vs. NYOKO = < 0.0001, NOKO vs. NYOKO = < 0.0001, degrees of freedom: 15, **Fig. EV1A** t ratio: SWAT = 5.17968, GWAT = 9.44711, BAT = 5.85914, *p* value: SWAT = 0.000075, GWAT = < 0.000001, BAT = 0.000019, degrees of freedom: 17, **Fig. EV1B** t ratio: day = 3.056, night = 3.453, total = 3.562, *p* value: day = 0.01568, night = 0.00865, total = 0.00738, degrees of freedom: 8, **Fig. EV1C** t ratio: day = 1.821, night = 0.212, total = 0.8141, *p* value: day = 0.1060, night = 0.8374, total = 0.4391, degrees of freedom: 8, **Fig. EV1D** t: 6-7 weeks = 0.4007, 17-18 weeks = 0.1752, *p* value: 6-7 weeks = 0.6934, 17-18 weeks = 0.8633, degrees of freedom: 6-7 weeks = 18, 17-18 weeks = 15, **Fig. EV1D** f value: 62.21, *p* value: WT vs. het NYKO = 0.5111, WT vs. NYKO = < 0.0001, het NYKO vs NYKO = < 0.0001, degrees of freedom: 37, **Fig. EV2B** t: cervical = 0.7674, lumbar = 1.163, *p* value: cervical = 0.4720, lumbar = 0.2888, degrees of freedom: 6, **Fig. EV2D** t ratio: ventro-lateral (cervical) = 3.062, ventro-lateral (lumbar) = 4.843, ventral (cervical) = 1.814, ventral (lumbar) = 1.4, *p* value: ventro-lateral (cervical) = 0.0376, ventro-lateral (lumbar) = 0.00838, ventral (cervical) = 0.1439, ventral (lumbar) = 0.2340, degrees of freedom: 4, **Fig. EV4E** f value: ventro-lateral (cervical) = 178.4, ventro-lateral (lumbar) = 625.8, ventral (cervical) = 164.3, ventral (lumbar) = 64.82, *p* value: ventro-lateral (cervical) = WT vs. NOKO 0.9704, WT vs. NYOKO = < 0.0001, NOKO vs. NYOKO = < 0.0001, ventro-lateral (lumbar) = WT vs. NOKO = 0.9460, WT vs. NYOKO = < 0.0001, NOKO vs. NYOKO = < 0.0001, ventral (cervical) = WT vs. NOKO = 0.8726, WT vs. NYOKO = < 0.0001, NOKO vs. NYOKO = < 0.0001, ventral (lumbar) = WT vs. NOKO = 0.9916, WT vs. NYOKO = 0.0001, NOKO vs. NYOKO = 0.0002, degrees of freedom: 6, **Fig. EV4F** t ratio: *Tnf*α = 4.651, *Il-6* = 0.2155, *Il-1*β = 5.371, *p* value: *Tnf*α =

0.00164, *Il-6* = 0.8347, *Il-1β* = 0.000669, degrees of freedom: 8, **Fig. EV5B** t ratio: moving mitochondria = 9.719, *p* value: moving mitochondria = 0.000627, degrees of freedom: 4. **Appendix Fig. S1A** t ratio: INL = 0.4762, ONL = 3.246, *p* value: INL = 0.6588, ONL = 0.0315, degrees of freedom: 4, **Appendix Fig. S1B** t ratio: 0.3984, *p* value: 0.7008, degrees of freedom: 8, **Appendix Fig. S1C** t ratio: *Hccs* = 1.551, *Cox7b* = 0.4014, *p* value: *Hccs* = 0.1595, *Cox7b* = 0.6986, degrees of freedom: 8, **Appendix Fig. S1E** t ratio: *Sod1* = 1.609, *Sod2* = 1.424, *Catalase* = 1.347, *Gpx1* = 1.096, *p* value: *Sod1* = 0.1462, *Sod2* =0.1924, *Catalase* = 0.2148, *Gpx1* = 0.3052, degrees of freedom: 8, **Appendix Fig.1G** t: 0.2043, *p* value: 0.8481 degrees of freedom: 4, **Appendix Fig. S2B** t ratio: *Tnfα* = 1.396, *Il-6* = 0.9848, *Il-1β* = 0.1314, *p* value: *Tnfα* = 0.2055, *Il-6* = 0.3576, *Il-1β* = 0.8992, degrees of freedom: 7, **Appendix Fig. S4D** t ratio: NDUFA9 = 2.673, SDHA = 0.6982, UQCRC2 SC = 4.702, UQCRC2 = 0.01006, MTCOX1 = 0.1735, ATP5a = 1.186, *p* value: NDUFA9 = 0.0282, SDHA = 0.5048, UQCRC2 SC = 0.0015, UQCRC2 = 0.9922, MTCOX1 = 0.8666, ATP5a = 0.2697, degrees of freedom: 8, **Appendix Fig S5A** t ratio: 10.48, *p* value: 0.0000046, degrees of freedom: 11, **Appendix Fig. S6E** t ratio: *Atg5* = 0.02324, *Beclin1* = 0.5217, *Atg7* = 0.3527, *Lc3b* = 0.184, *Bnip3* = 1.328, *Bnip3l* = 0.3965, *p* value: *Atg5* = 0.9820, *Beclin1* = 0.6160, *Atg7* = 0.7334, *Lc3b* = 0.8586, *Bnip3* = 0.2207, *Bnip3l* = 0.7021, degrees of freedom: 8.
