# Supplementary material for: Loss of the mitochondrial i‐AAA protease YME1L leads to ocular dysfunction and spinal axonopathy
Source: EMBO Mol Med. 2018 Nov 2;11(1):e9288. doi: 10.15252/emmm.201809288 (PMC6328943; doi:10.15252/emmm.201809288)
Supplement: Supplementary file 2 — Expanded View Figures PDF [file EMMM-11-e9288-s002.pdf]

Expanded View Figures

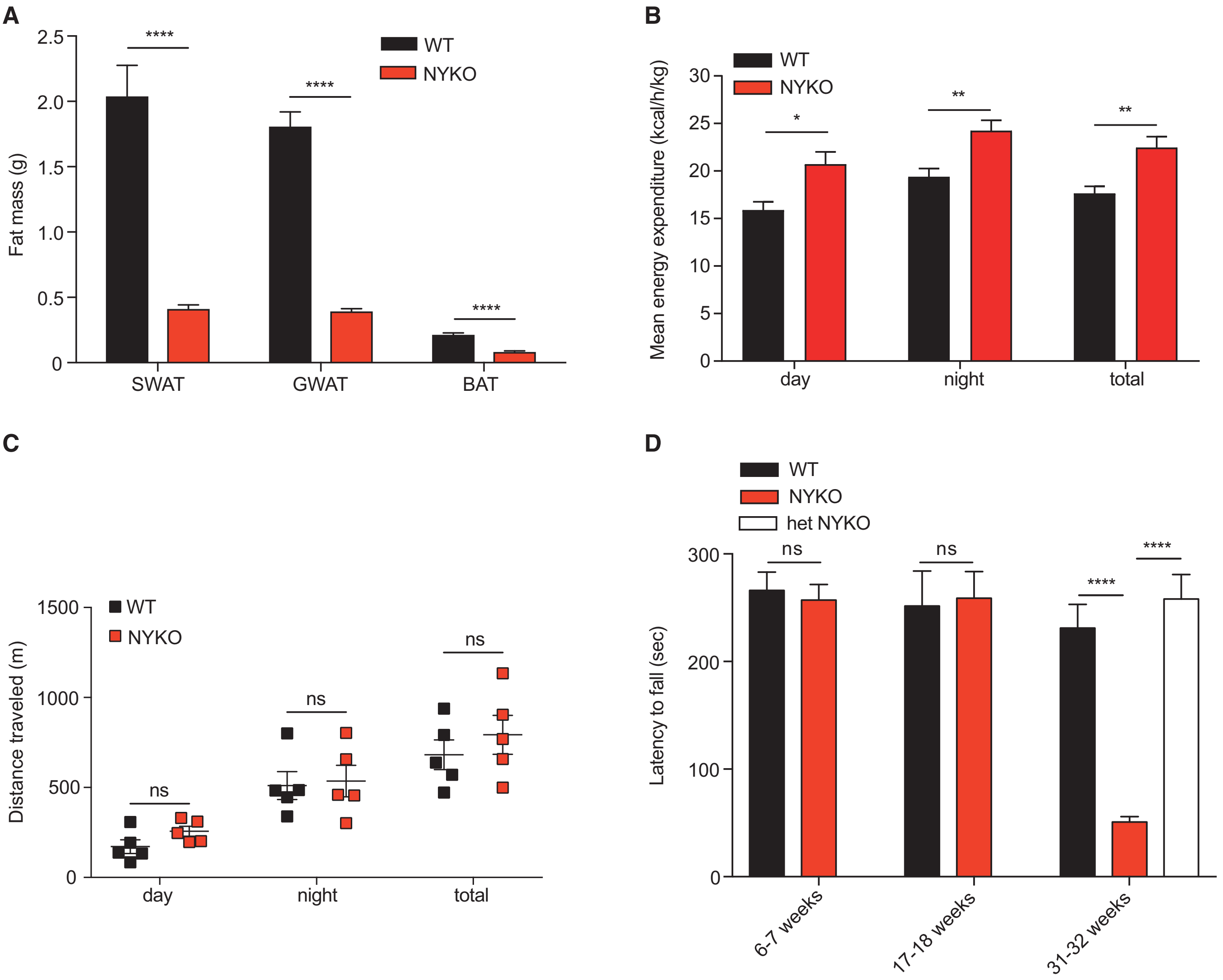

Figure EV1. Phenotypic characterization of NYKO mice.

A Fat pad mass of subcutaneous white adipose tissue (SWAT), gonadal white adipose tissue (GWAT), and brown adipose tissue (BAT) isolated from 31- to 32-week-old WT (n = 12) and NYKO (n = 7) male mice.

B Mean energy expenditure of WT (n = 5) and NYKO (n = 5) male mice after disease onset at 26 weeks of age.

C Day and night activity of 26-week-old, male WT (n = 5) and NYKO (n = 5) mice.

D Wire mesh grip strength test of 6- to 7-week-old WT (n = 10) and NYKO mice (n = 10), 17- to 18-week-old WT (n = 8) and NYKO mice (n = 9), and 31- to 32-week-old mice WT (n = 12), NYKO (n = 19), and heterozygous NYKO mice (het NYKO, n = 9).

Data information: Unpaired t-test was used for comparison of two groups, ordinary one-way ANOVA for comparison of three groups. *$P \leq 0.05$, **$P \leq 0.01$, ****$P \leq 0.0001$, ns = not significant. Data are means ± SEM.

© 2018 The Authors

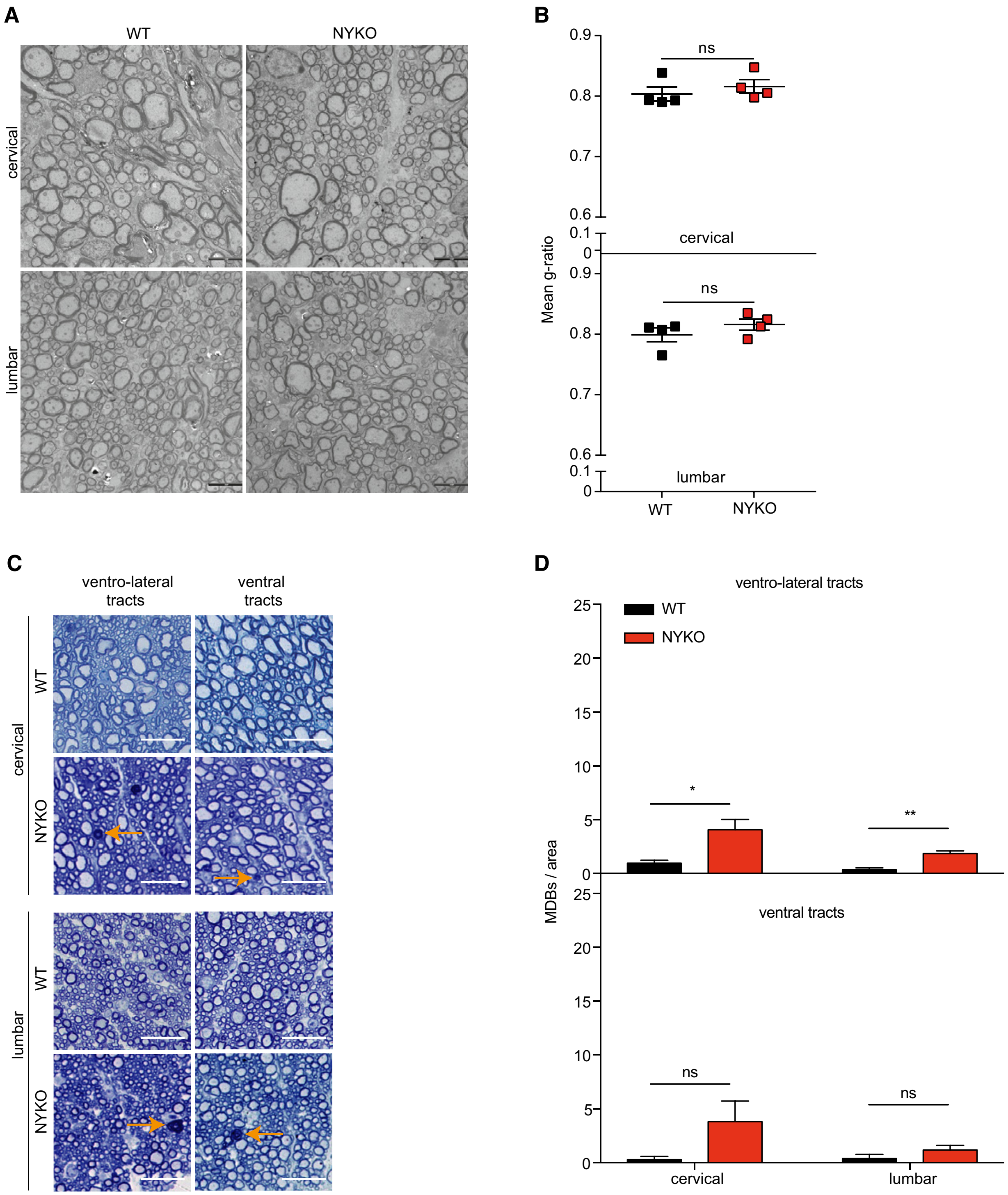

Figure EV2. Mild neurodegeneration in ventro-lateral spinal cord tracts of aged NYKO mice.

A TEM analysis of dorso-lateral tracts in spinal cord sections of 6- to 7-week-old WT and NYKO mice. Scale bars, 5 μm.

B Quantification of g-ratios from 200 fibers per genotype (n = 4).

C Transverse semithin sections of spinal cords of WT and NYKO mice. Sections were stained with toluidine blue. Orange arrows point to myelin dense bodies (MDBs) indicating degenerating neurons. Scale bars, 25 μm.

D MDBs in ventro-lateral and ventral tracts of spinal cords of 31- to 32-week-old WT (n = 3) and NYKO (n = 3) mice. Scale bar, 500 μm.

Data information: Unpaired t-test, *$P \leq 0.05$, **$P \leq 0.01$, ns = not significant. Data are means ± SEM.

 © 2018 The Authors

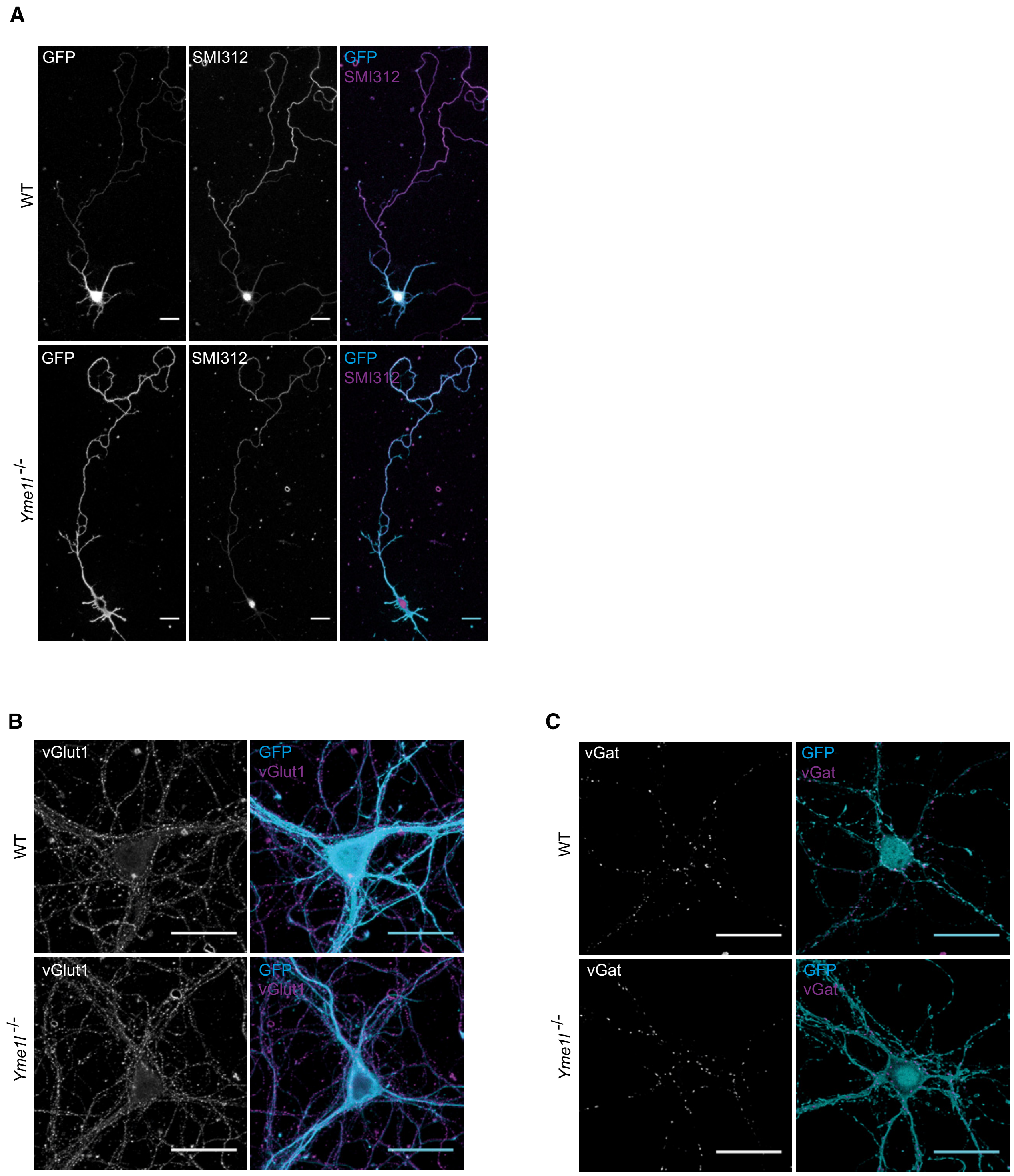

Figure EV3. YME1L is dispensable for neuronal development and synapse formation.

A Fluorescence images of isolated cortical neurons from *Yme1l*$^{fl/fl}$ animals expressing CAG-GFP (WT) or CAG-Cre-IRES-GFP (*Yme1l*$^{-/-}$) at DIV7 and stained with SMI312 to label axons. Scale bars, 30 µm.

B, C Fluorescence images of isolated cortical neurons from *Yme1l*$^{fl/fl}$ animals expressing CAG-GFP (WT) or CAG-Cre-IRES-GFP (*Yme1l*$^{-/-}$) at DIV12 and stained with (B) vesicular glutamate transporter 1 (vGlut1) or (C) vesicular GABA transporter (vGat) to label excitatory and inhibitory synapses, respectively. Scale bars, 30 µm.

Source data are available online for this figure.

Figure EV4. Ablation of *Oma1* in NYKO mice does not restore retinal organization and causes axonal degeneration in ventro-lateral and ventral tracts of the spinal cord.

A Immunoblot analysis of spinal cord lysates from 6- to 7-week-old WT, NYKO, NOKO, and NYOKO mice (n = 3) using the indicated antibodies. SDHA was used as a loading control. * indicates unspecific antibody binding.

B, C Retinal sagittal cross sections from 6- to 7-week-old NYOKO mice (n = 4) stained with hematoxylin and eosin. NFL = nerve fiber layer, IPL = inner plexiform layer, INL = inner nuclear layer, OPL = outer plexiform layer, ONL = outer nuclear layer, R&C = rods and cones. Scale bar, 30 μm.

D Transverse semithin sections of spinal cords of 6- to 7-week-old WT, NOKO, and NYOKO mice. Sections were stained with toluidine blue. Orange arrows point to MDBs indicating degenerating neurons. Scale bars, 25 μm.

E MDBs in ventro-lateral and ventral tracts of 6- to 7-week-old WT (n = 3), NOKO (n = 3), and NYOKO (n = 3) mice.

F mRNA levels of proinflammatory cytokines from 6- to 7-week-old retinas (WT, n = 5; NYOKO, n = 5). Transcript levels were normalized to *Hprt* mRNA levels.

Data information: Unpaired t-test was used for comparison of two groups, ordinary one-way ANOVA for comparison of three groups. $^{**}P \leq 0.01$, $^{***}P \leq 0.001$, $^{****}P \leq 0.0001$, ns = not significant. Data are means ± SEM.

Source data are available online for this figure.

 © 2018 The Authors

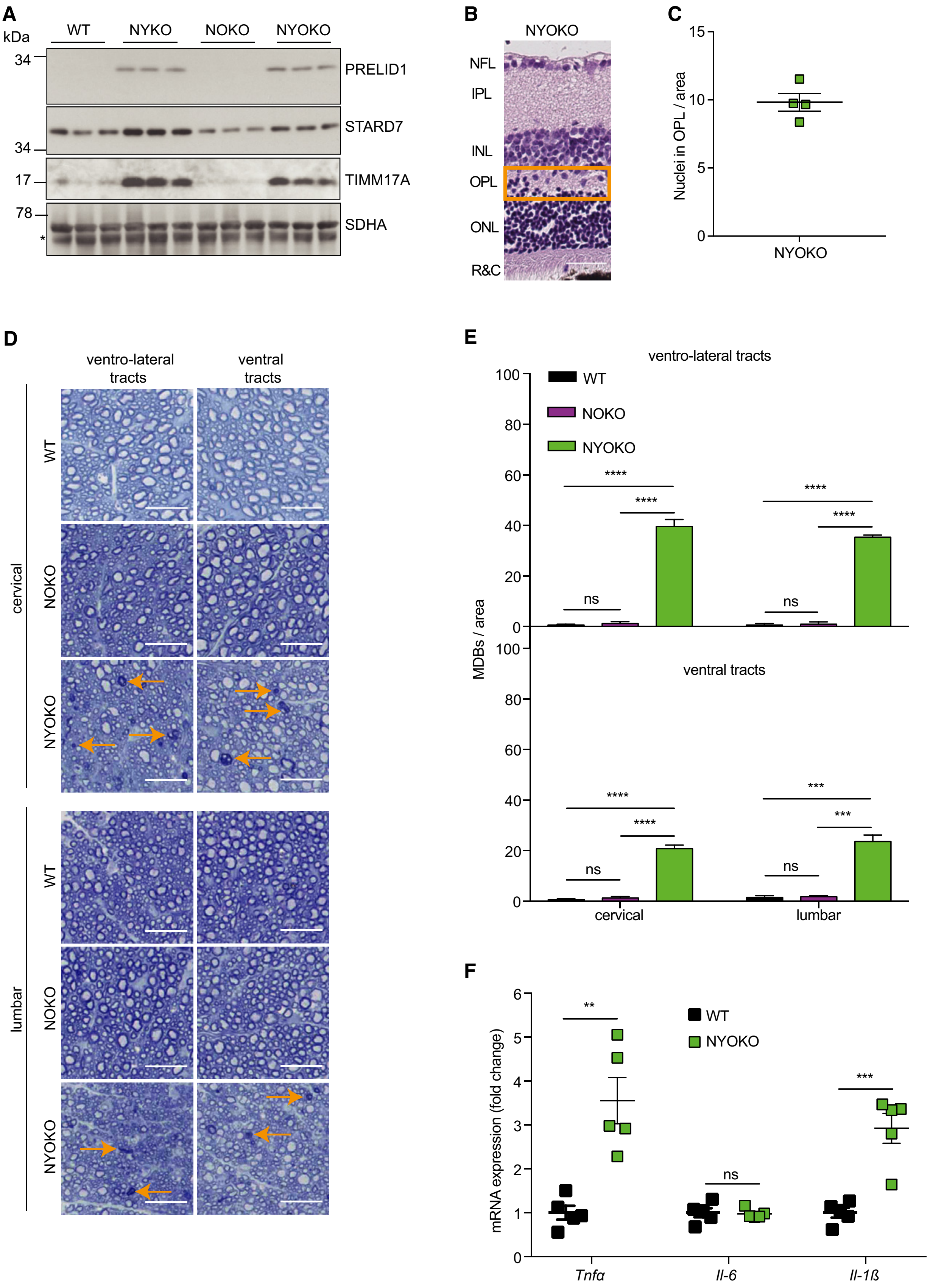
A
kDa
WT
NYKO
NOKO
NYOKO
34
PRELID1
STARD7
34
17
TIMM17A
78
*
SDHA
B
NYOKO
NFL
IPL
INL
OPL
ONL
R&C
C
Nuclei in OPL / area
15
10
5
0
NYOKO
D
ventro-lateral tracts
ventral tracts
cervical
lumbar
WT
NOKO
NYOKO
E
ventro-lateral tracts
ventral tracts
WT
NOKO
NYOKO
MDBs / area
100
80
60
40
20
0
****
ns
***
cervical
lumbar
F
mRNA expression (fold change)
6
5
4
3
2
1
0
**
ns
***
WT
NYOKO
Tnfα
Il-6
Il-1ß

Figure EV4.

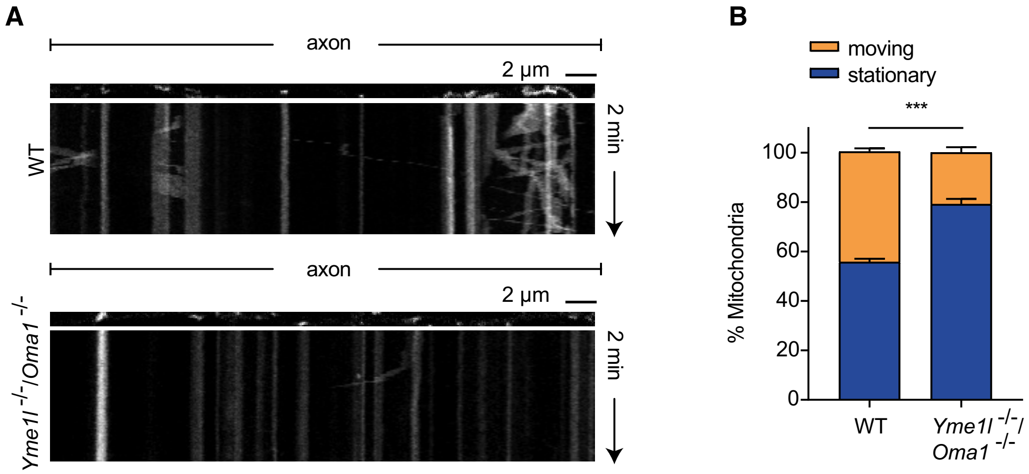

Figure EV5. Loss of OMA1 does not restore axonal trafficking of mitochondria in the absence of YME1L.

A, B Kymographs showing mitochondrial movement in representative axons. Vertical lines correspond to stationary mitochondria and diagonal lines to moving mitochondria. Cortical neurons were isolated from *Yme1l*$^{fl/fl}$/*Oma*$^{fl/fl}$ animals and transfected with CAG-GFP- (WT) or CAG-GFP-IRES-Cre (*Yme1l*$^{-/-}$/*Oma1*$^{-/-}$) expressing plasmids. Mitochondria were stained using Mitotracker. In total, 18 axonal segments were analyzed for WT control neurons and 18 axonal segments for *Yme1l*$^{-/-}$/*Oma1*$^{-/-}$ neurons. Data are presented as means of three independent experiments ± SEM. Unpaired *t*-test, ***$P \leq 0.001$.

© 2018 The Authors
