# Supplementary material for: Loss of the mitochondrial i‐AAA protease YME1L leads to ocular dysfunction and spinal axonopathy
Source: EMBO Mol Med. 2018 Nov 2;11(1):e9288. doi: 10.15252/emmm.201809288 (PMC6328943; doi:10.15252/emmm.201809288)
Supplement: Supplementary file 3 — Movie EV1 [file EMMM-11-e9288-s003.zip › Movie_EV1/Movie_EV1_Legend.docx]

Movie EV1 Legend: WT 31-32 wks
