# Supplementary material for: Loss of the mitochondrial i‐AAA protease YME1L leads to ocular dysfunction and spinal axonopathy
Source: EMBO Mol Med. 2018 Nov 2;11(1):e9288. doi: 10.15252/emmm.201809288 (PMC6328943; doi:10.15252/emmm.201809288)
Supplement: Supplementary file 5 — Movie EV3 [file EMMM-11-e9288-s005.zip › Movie_EV3/Movie_EV3_Legend.docx]

Movie EV3 Legend: WT Neurons Yme1l fl GFP Mitotracker.
