# Supplementary material for: Loss of the mitochondrial i‐AAA protease YME1L leads to ocular dysfunction and spinal axonopathy
Source: EMBO Mol Med. 2018 Nov 2;11(1):e9288. doi: 10.15252/emmm.201809288 (PMC6328943; doi:10.15252/emmm.201809288)
Supplement: Supplementary file 6 — Movie EV4 [file EMMM-11-e9288-s006.zip › Movie_EV4/Movie_EV4_Legend.docx]

Movie EV4 Legend: YKO Neurons Yme1l fl CreGFP Mitotracker.
