# Supplementary figures and images for: Loss of the mitochondrial i‐AAA protease YME1L leads to ocular dysfunction and spinal axonopathy
Source: EMBO Mol Med. 2018 Nov 2;11(1):e9288. doi: 10.15252/emmm.201809288 (PMC6328943; doi:10.15252/emmm.201809288)

1D

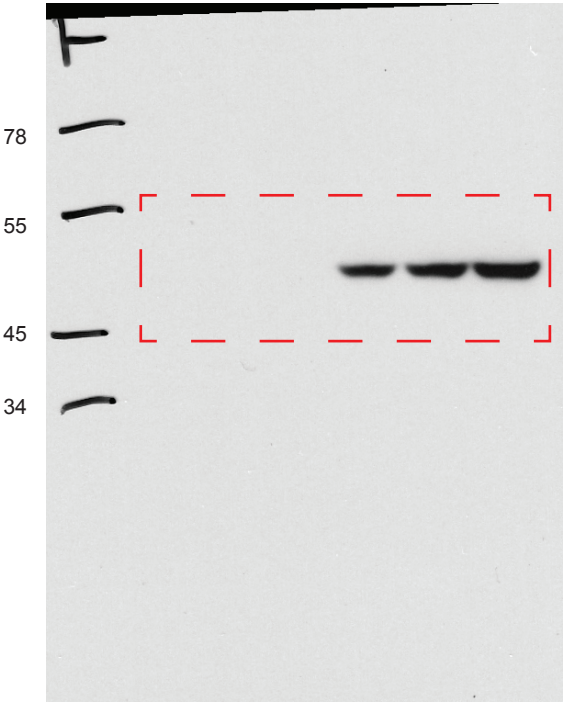

GFAP

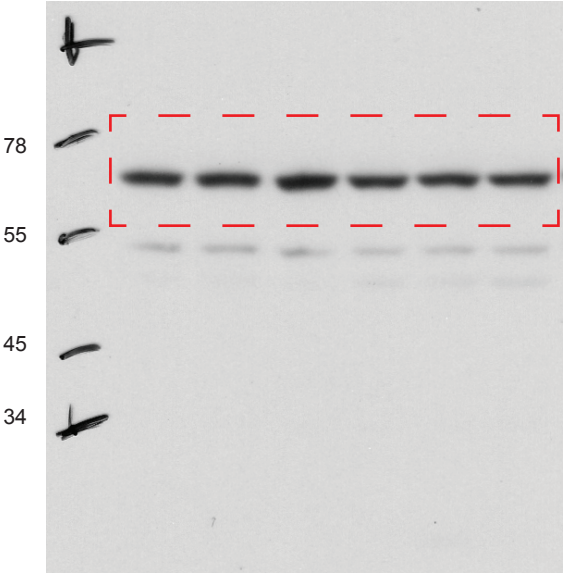

SDHA

Supplement: Supplementary file 9 — Source Data for Figure 1 [file EMMM-11-e9288-s007.pdf]

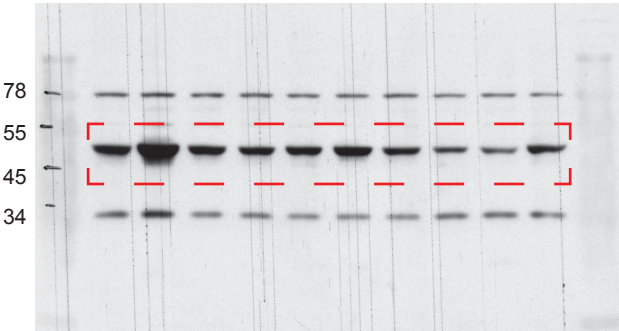

FB,GFAP

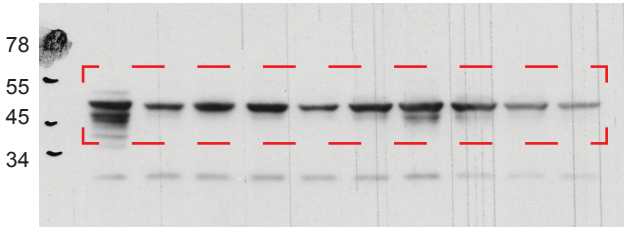

CB,GFAP

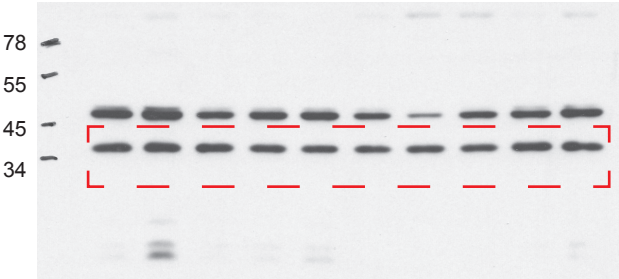

FB,GAPDH

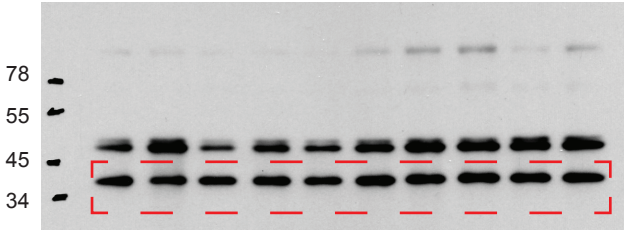

CB,GAPDH

Supplement: Supplementary file 10 — Source Data for Figure 3 [file EMMM-11-e9288-s008.pdf]

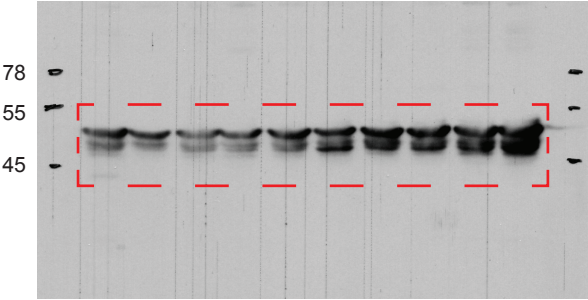

GFAP

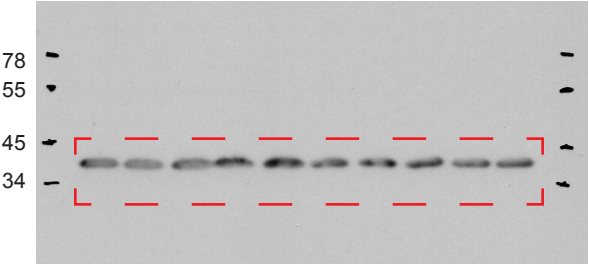

GAPDH

Supplement: Supplementary file 11 — Source Data for Figure 4 [file EMMM-11-e9288-s009.pdf]

5A

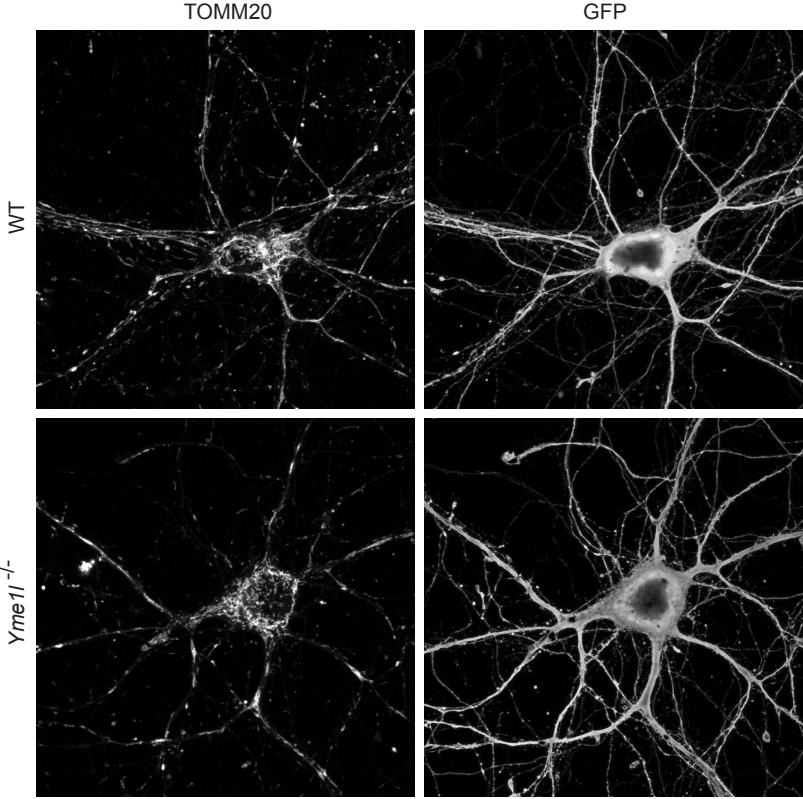

Supplement: Supplementary file 12 — Source Data for Figure 5 [file EMMM-11-e9288-s010.pdf]

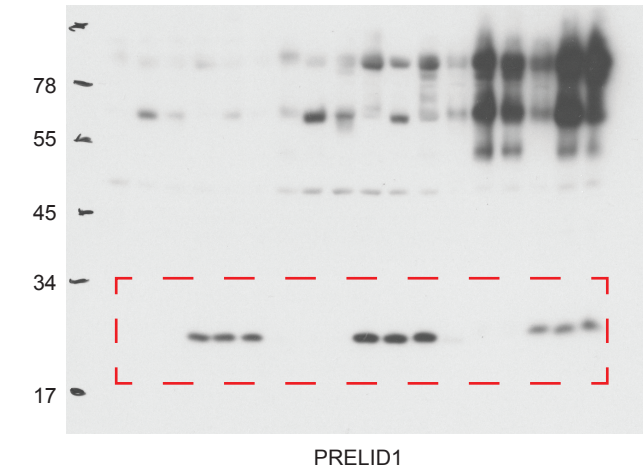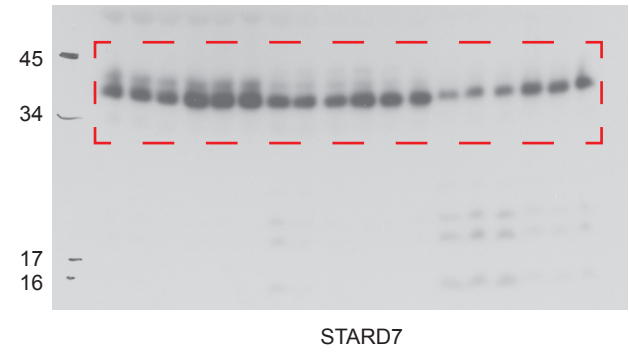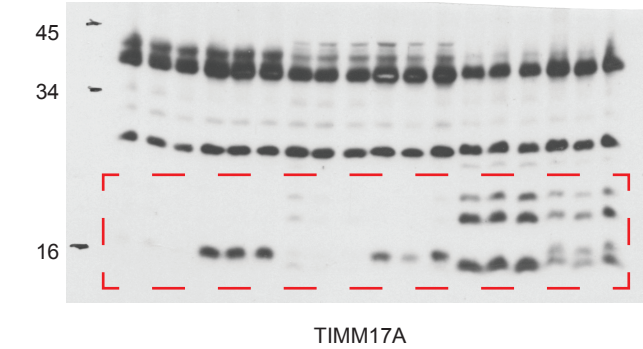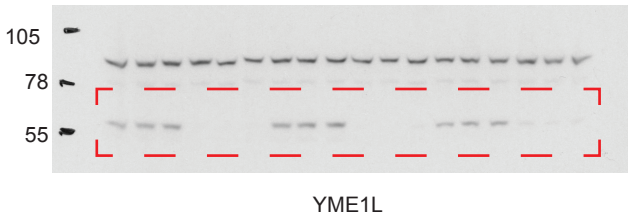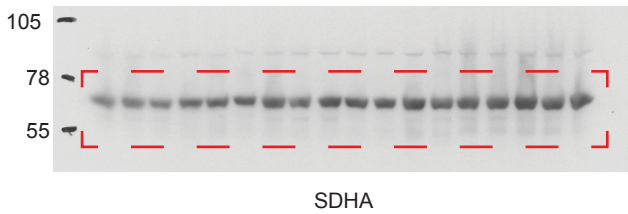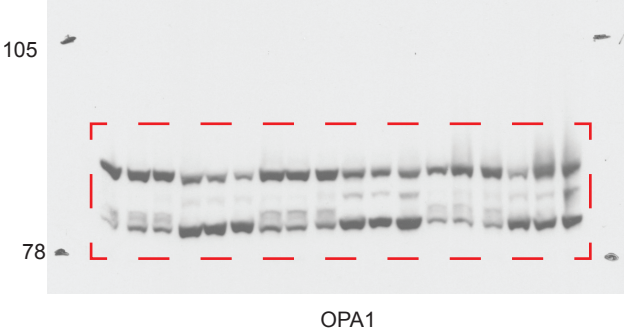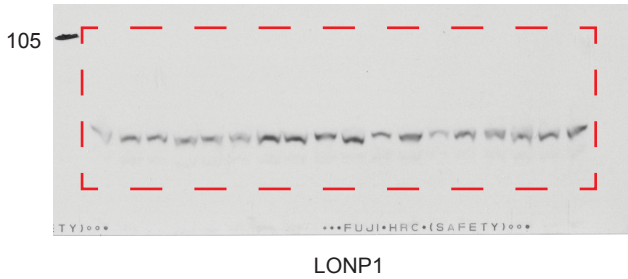

Supplement: Supplementary file 13 — Source Data for Figure 7 [file EMMM-11-e9288-s011.pdf]

8A

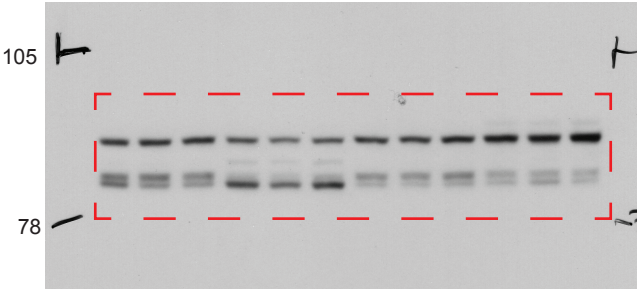

Retina, OPA1

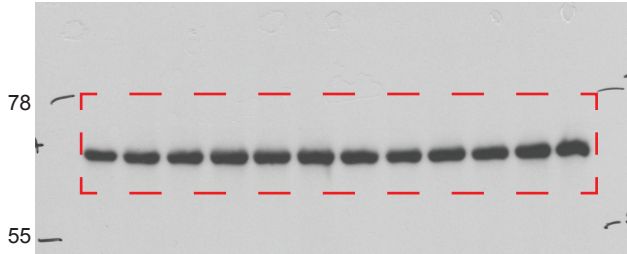

Retina, SDHA

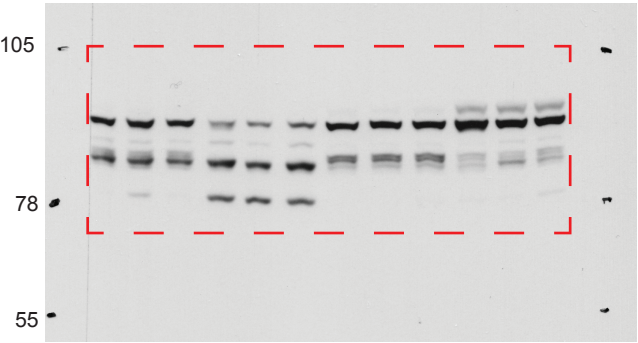

Spinal cord, OPA1

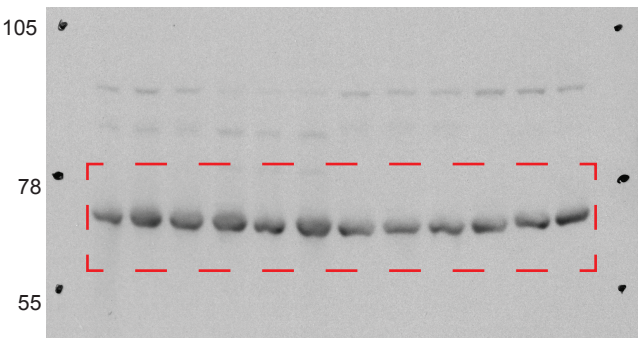

Spinal cord, SDHA

8D

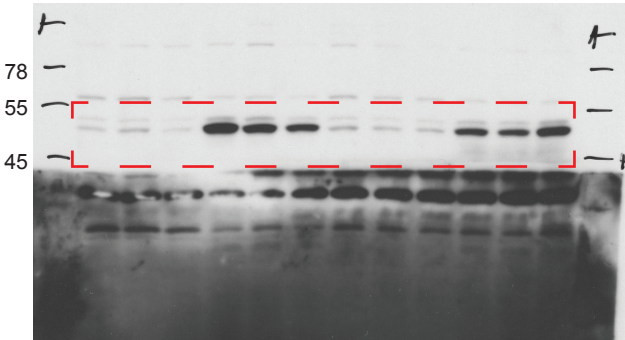

Retina, GFAP

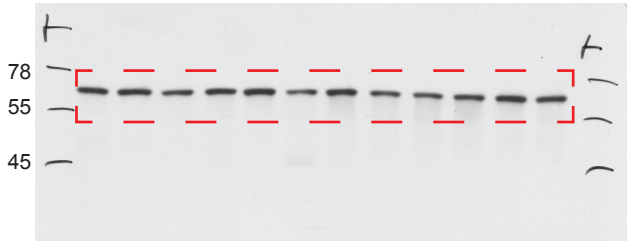

Retina, SDHA (2)

Supplement: Supplementary file 14 — Source Data for Figure 8 [file EMMM-11-e9288-s012.pdf]
