# Supplementary material for: Loss of the mitochondrial i‐AAA protease YME1L leads to ocular dysfunction and spinal axonopathy
Source: EMBO Mol Med. 2018 Nov 2;11(1):e9288. doi: 10.15252/emmm.201809288 (PMC6328943; doi:10.15252/emmm.201809288)
Supplement: Supplementary file 7 — Source Data for Expanded View and Appendix [file EMMM-11-e9288-s013.zip › emmm201809288-sup-0013-SDataAppendixFigs.zip!/Source_Data_for_Appendix_Figures/Sprenger_et_al_SDataAppendixFigS4.pdf]

4A

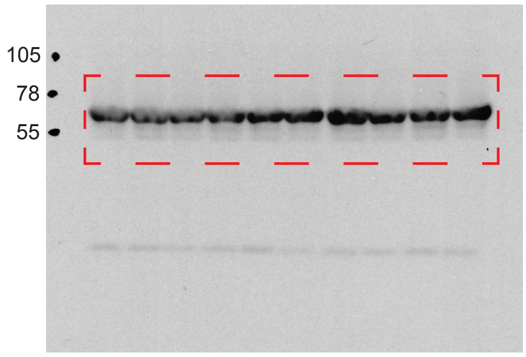

SDHA

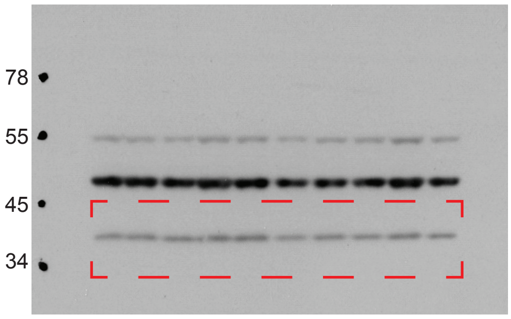

NDUFA9

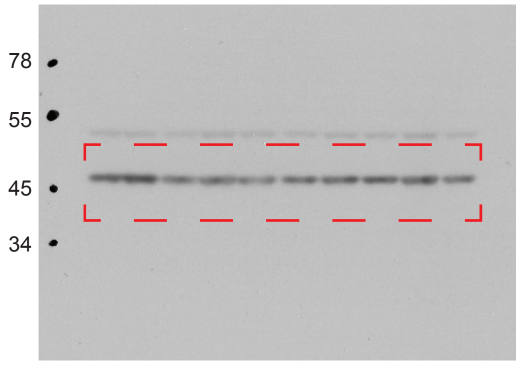

UQCRC2

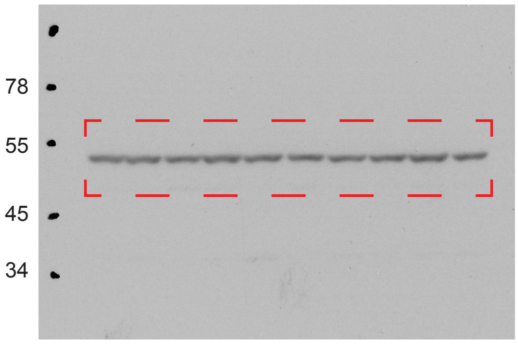

ATP5a

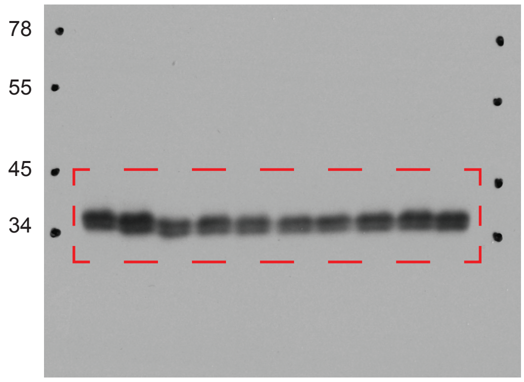

MTCO1

4B

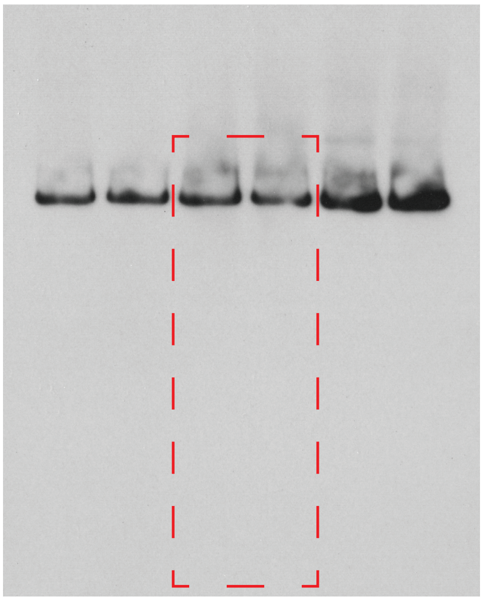

NDUFA9

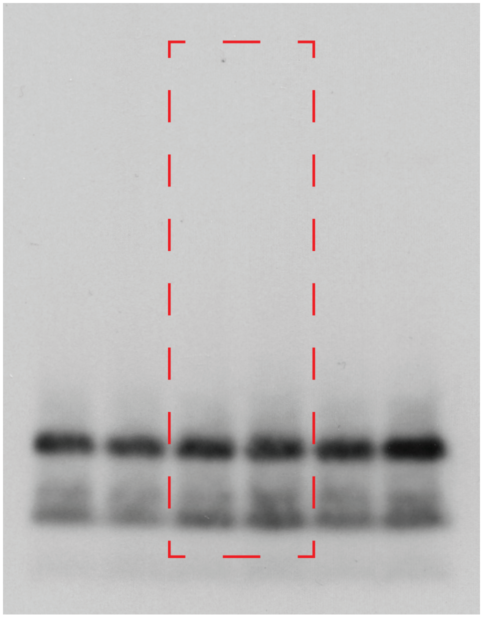

SDHA

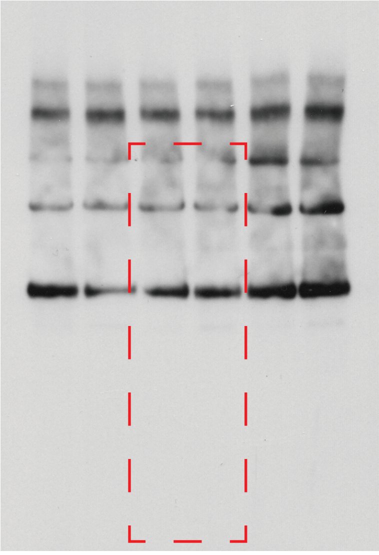

UQCRC2

SDataAppendixFigS4

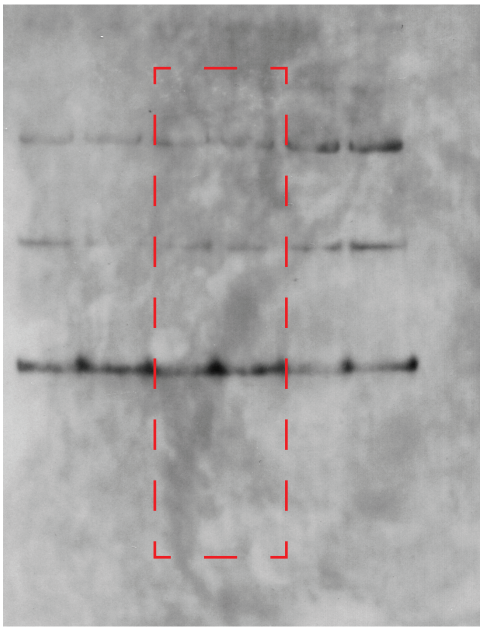

MTCO1

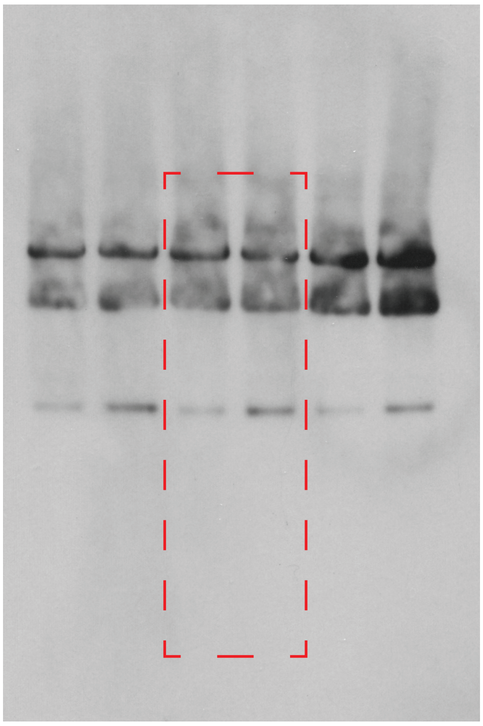

ATP5a

4C

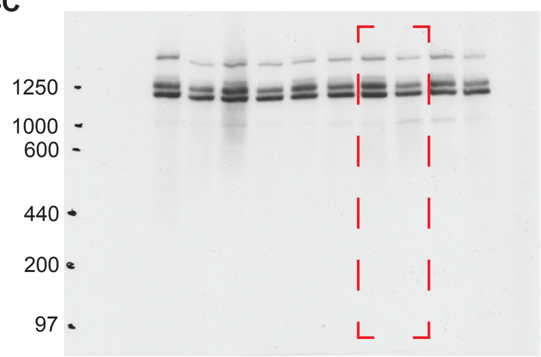

NDUFA9

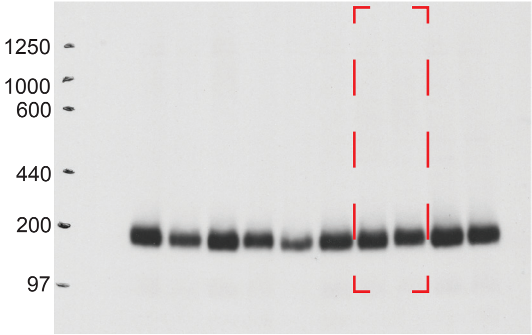

SDHA

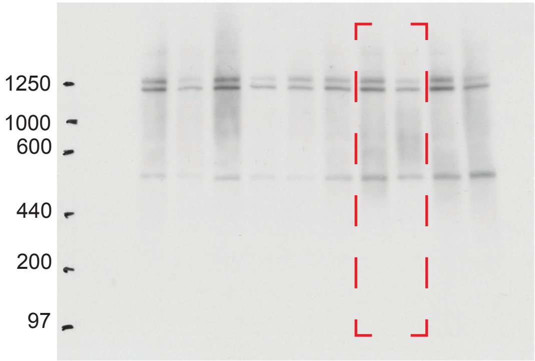

UQCRC2

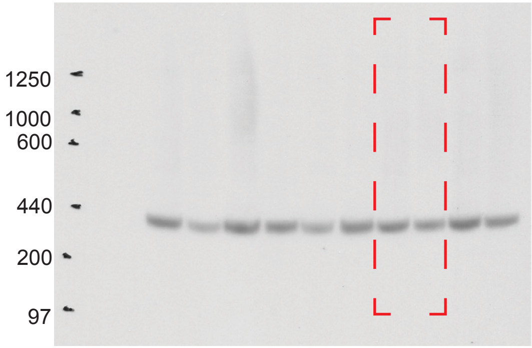

MTCO1

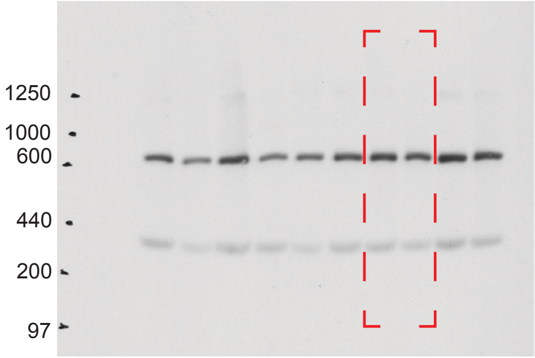

ATP5a
