# Supplementary material for: Loss of the mitochondrial i‐AAA protease YME1L leads to ocular dysfunction and spinal axonopathy
Source: EMBO Mol Med. 2018 Nov 2;11(1):e9288. doi: 10.15252/emmm.201809288 (PMC6328943; doi:10.15252/emmm.201809288)
Supplement: Supplementary file 7 — Source Data for Expanded View and Appendix [file EMMM-11-e9288-s013.zip › emmm201809288-sup-0013-SDataAppendixFigs.zip!/Source_Data_for_Appendix_Figures/Sprenger_et_al_SDataAppendixFigS5.pdf]

5B

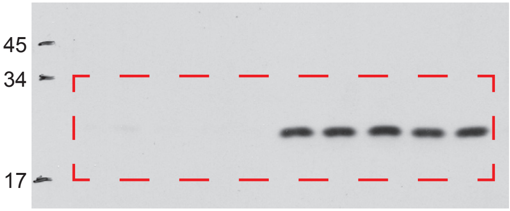

PRELID1

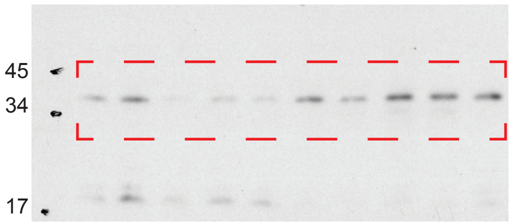

STARD7

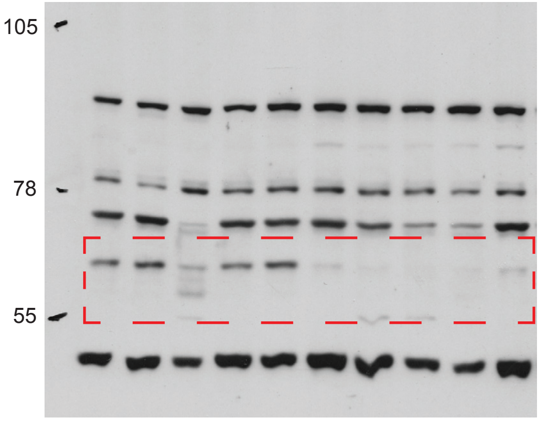

YME1L

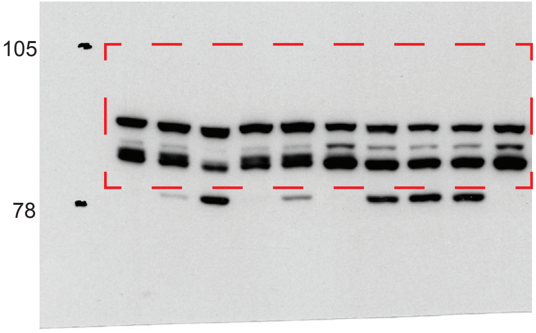

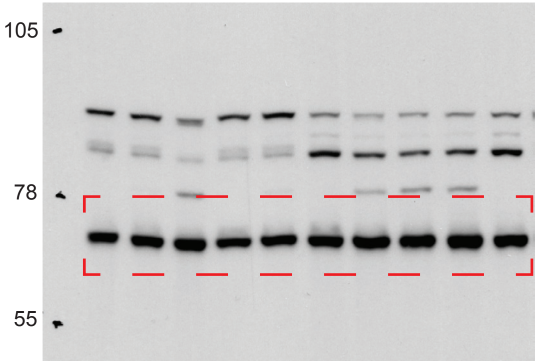

SDHA

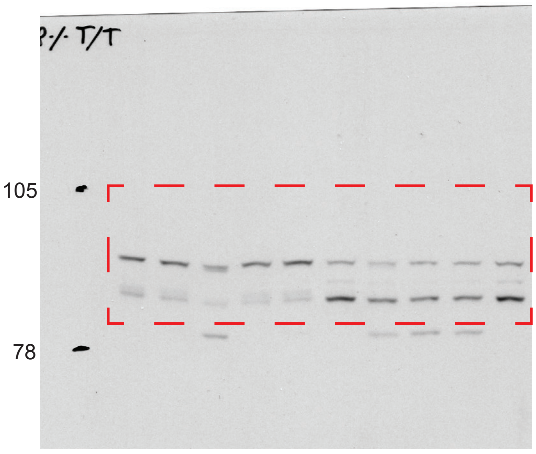

OPA1

5C

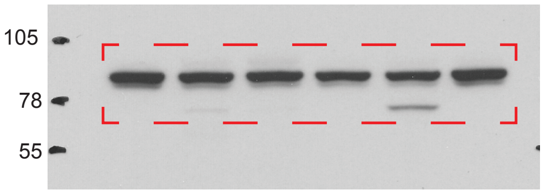

DRP1

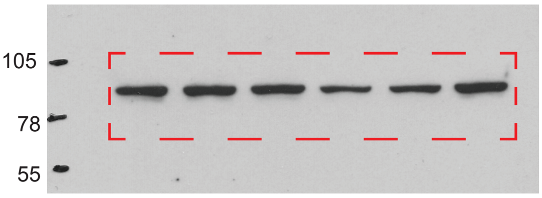

pDRP1S616

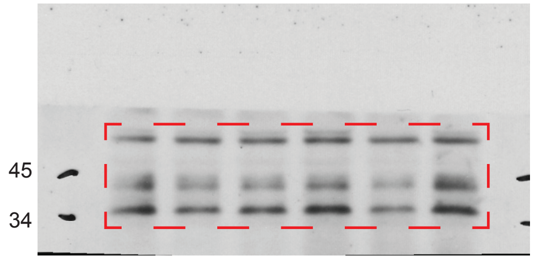

MFF

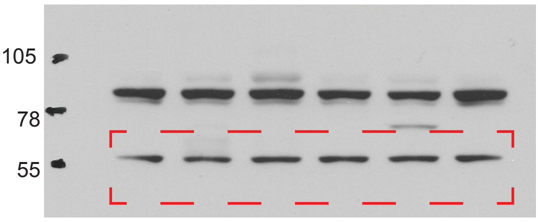

HSP60

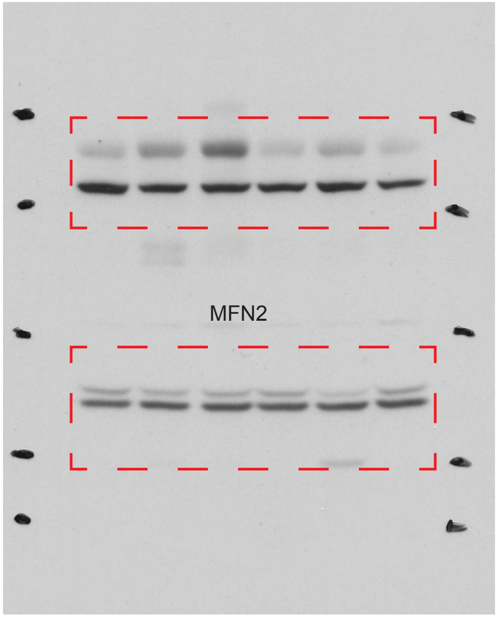

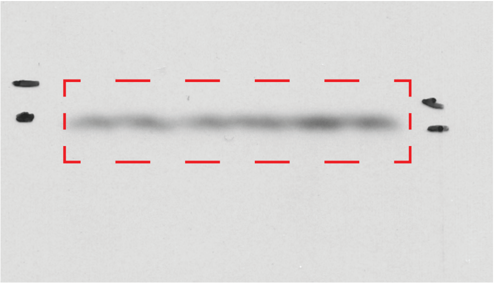

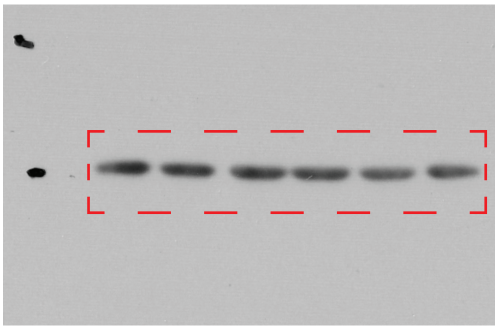

SDataAppendixFigS5
