# Supplementary material for: Loss of the mitochondrial i‐AAA protease YME1L leads to ocular dysfunction and spinal axonopathy
Source: EMBO Mol Med. 2018 Nov 2;11(1):e9288. doi: 10.15252/emmm.201809288 (PMC6328943; doi:10.15252/emmm.201809288)

3A

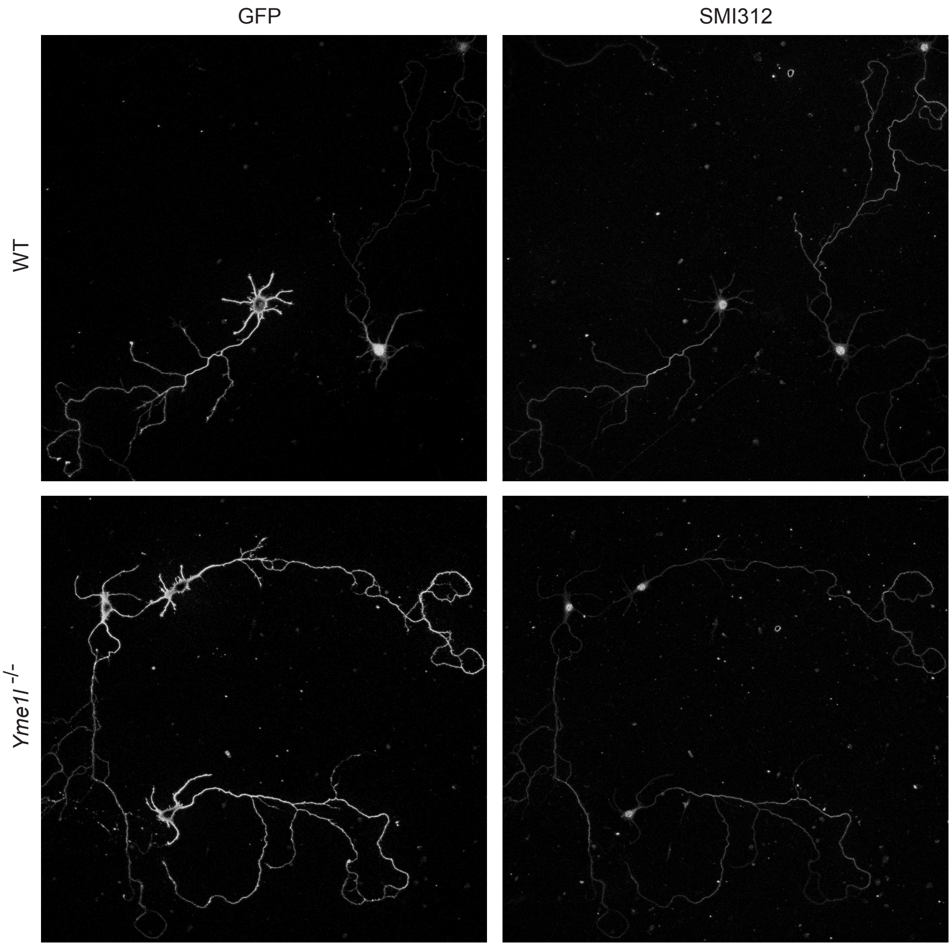

3B

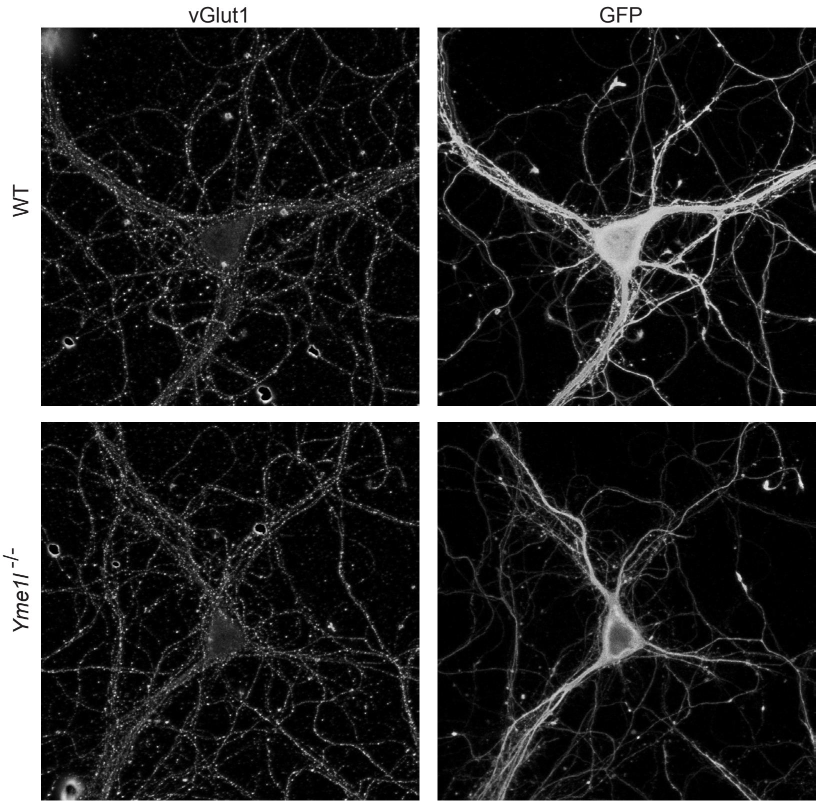

3C

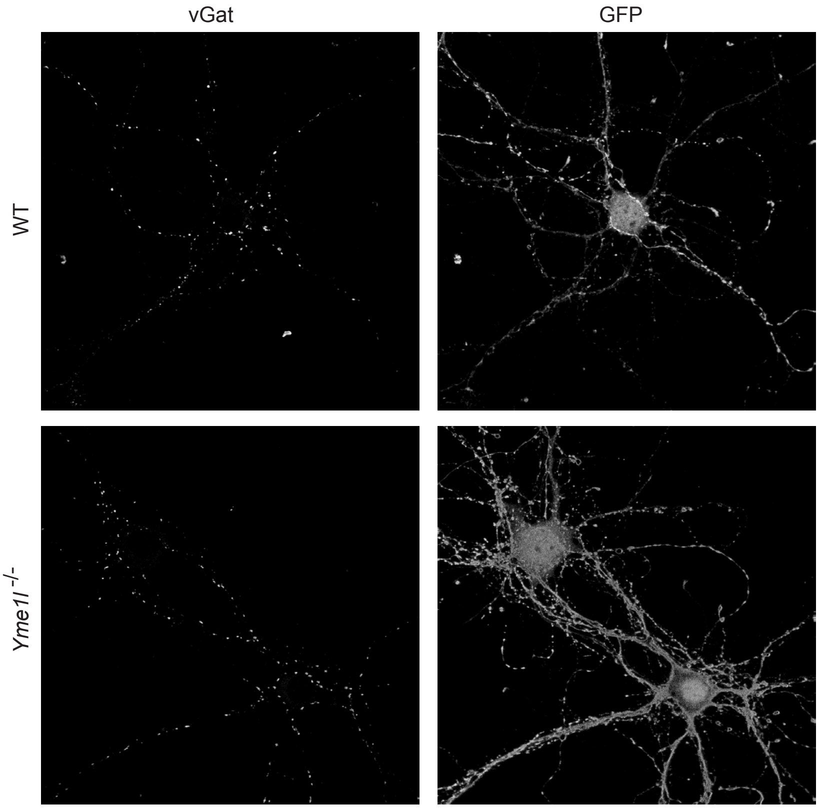

Supplement: Supplementary file 7 — Source Data for Expanded View and Appendix [file EMMM-11-e9288-s013.zip › emmm201809288-sup-0014-SDataEVFigs.zip!/Source_Data_for_EV_Figures/Sprenger_et_al_SDataFigEV3.pdf]
